# The psychological and behavioural factors associated with laypeople initiating CPR for out-of-hospital cardiac arrest: a systematic review

**DOI:** 10.1186/s12872-022-02904-2

**Published:** 2023-01-14

**Authors:** Barbara Farquharson, Diane Dixon, Brian Williams, Claire Torrens, Melanie Philpott, Henriette Laidlaw, Siobhan McDermott

**Affiliations:** 1 NMAHP Research Unit, University of Stirling, Stirling, FK9 4LA UK; 2 University of Aberdeen, Aberdeen, UK; 3 University of Highlands and Islands, Inverness, UK; 4 University of Stirling, Stirling, UK; 5 Independent Contract Researcher, Stirling, UK

**Keywords:** CPR, Bystander, Laypeople, Systematic review, Psychological, Behavioural, Out-of-hospital cardiac arrest

## Abstract

**Background:**

Prompt, effective CPR greatly increases the chances of survival in out-of-hospital c ardiac arrest. However, it is often not provided, even by people who have previously undertaken training. Psychological and behavioural factors are likely to be important in relation to CPR initiation by lay-people but have not yet been systematically identified.

**Methods:**

Aim: to identify the psychological and behavioural factors associated with CPR initiation amongst lay-people.

Design: Systematic review

Data sources: Cochrane Library, MEDLINE, EMBASE, CINAHL, PsycInfo and Google Scholar.

Study eligibility criteria: Primary studies reporting psychological or behavioural factors and data on CPR initiation involving lay-people published (inception to 31 Dec 2021).

Study appraisal and synthesis methods: Potential studies were screened independently by two reviewers. Study characteristics, psychological and behavioural factors associated with CPR initiation were extracted from included studies, categorised by study type and synthesised narratively.

**Results:**

One hundred and five studies (150,820 participants) comprising various designs, populations and of mostly weak quality were identified. The strongest and most ecologically valid studies identified factors associated with CPR initiation: the *overwhelming emotion of the situation*, *perceptions of capability*, *uncertainty about when CPR is appropriate*, *feeling unprepared* and *fear of doing harm*. Current evidence comprises mainly atheoretical cross-sectional surveys using unvalidated measures with relatively little formal testing of relationships between proposed variables and CPR initiation.

**Conclusions:**

Preparing people to manage strong emotions and increasing their perceptions of capability are likely important foci for interventions aiming to increase CPR initiation. The literature in this area would benefit from more robust study designs.

**Systematic review registration:**

PROSPERO: CRD42018117438.

**Supplementary Information:**

The online version contains supplementary material available at 10.1186/s12872-022-02904-2.

## Introduction

Out of hospital cardiac arrest (OHCA) has a devastatingly high mortality rate [1]. Survival to hospital discharge ranges between countries from < 1% [2] to 25% in the best European centres [3], reflecting differences in case identification, demography, geography and emergency service provision [4]. Reducing the mortality associated with OHCA is a strategic priority of many countries [5–10].

Prompt, effective bystander cardiopulmonary resuscitation (CPR) is the most important factor determining survival from OHCA, increasing survival almost 4-fold [11, 12]. Registry data show most OHCA occur at home [2, 13, 14]. Even the most prompt emergency medical response will take at least a few minutes (median 6 mins.) [15], and so the response of others in the home is critical.

Governments and charities invest significantly in training lay-people in CPR [16–18]. Despite this, those in OHCA often do not receive CPR prior to the arrival of emergency services [19]. Even amongst those who are trained, less than half attempt CPR when required [20]. Increasing the *proportion* of lay-people trained in CPR who actually apply their skills in a real emergency situation is essential [21] as otherwise much of the effort expended in training lay-people will not improve outcomes for patients.

Research relating to CPR training of lay-people has largely been concerned with increasing knowledge and achieving competence in the skill of CPR. Questions of how best to teach CPR tend to be answered by studies using skills performance (e.g. compression depth) and assessment of knowledge as outcome measures [22, 23]. However the International Liaison Committee on Resuscitation [24] and behavioural science [25] would suggest that psychological factors (e.g. people’s attitudes about CPR) are likely to be critical in explaining whether or not people initiate CPR. To date there has not been a systematic synthesis of this literature.

The aim of this review was to synthesise evidence relating to lay-people initiating CPR and to identify the psychological and behavioural factors that facilitate or inhibit people’s willingness to perform CPR.

## Method

### Protocol and registration

In line with best practice, a review protocol was published (2018) and registered with the PROSPERO International Prospective Register of systematic reviews (protocol number 117438): https://www.crd.york.ac.uk/PROSPERO/display_record.php?RecordID=117438.

### Eligibility criteria

#### Inclusion

##### Types of study

All primary study designs.

##### Types of participants

Lay members of the public (i.e. not healthcare professionals or others who receive CPR training as a part of their job, e.g. lifeguards) of any age.

##### Types of outcome measure

Studies which contained psychological/behavioural data (not CPR knowledge or training status) related to 1) why the participants did or did not perform CPR in real emergencies or 2) would or would not perform CPR in a hypothetical or simulated situation. CPR was defined as performing chest compressions (CC), mouth-to-mouth ventilations, applying an Automated External Defibrillator (AED) or any combination of these.

#### Exclusion

Papers which did not report a primary empirical study (e.g. reviews, editorials, opinion pieces) were excluded.

### Information sources and search strategy

Six electronic databases - Cochrane Library, MEDLINE, EMBASE, CINAHL, PsycInfo and Google Scholar- were searched for publications from inception of each database to 13th December 2019 (search strategy is supplied in supplementary materials Additional file 1). Supplementary searches included: a) reference lists of included studies, b) citations of included studies (Science Citation Index (SCI), Social Sciences Citation Index (SSCI) and Arts and Humanities Citation Index (A&HCI), c) hand-searches of titles (Jan 2005 – Jan 2020) of *Resuscitation* and a further update database search performed 01/06/21.

### Study selection

Screening of titles was undertaken independently by two reviewers (BF and DD) to exclude titles that were obviously irrelevant. The inclusion/exclusion criteria were applied to abstracts of studies and irrelevant abstracts were excluded. Inter-rater agreement kappa was 0.85, pabak kappa = 0.85. Full texts considered potentially relevant by either reviewer were screened independently (BF and DD). At full-text stage, any disagreements between the reviewers were resolved by discussion.

### Assessment of methodological quality and risk of bias

The methodological quality of studies was assessed using the Effective Public Health Practice Project (EPHPP) Quality Assessment Tool for quantitative studies [26] and the Joanna Briggs Institute’s Quality Assessment and Review Instrument (QARI) for qualitative studies [27]. Included studies were independently assessed by two reviewers (BF and CT) for methodological quality, with discrepancies being resolved through discussion.

### Data extraction

Guided by the CONSORT guidelines [28] and the published protocol, the following data were extracted for each study: study details (author & date, location, study duration, objectives), study methods (design, setting, target population, sample size estimation, actual sample size, sampling and recruitment method, behavioural and psychological data, analysis, dates of recruitment) and study results.

BF and SM independently performed data extraction on 20% of the included studies (*n* = 20) to assess reliability. No discrepancies in independently extracted data were found and the remainder were extracted by a single researcher (BF or SM).

### Synthesis and analysis

Behavioural and psychological factors identified during extraction were grouped into conceptually similar ‘factors’ by BF: 51 individual factors were identified. To facilitate interpretation, this large number of factors were grouped using categorisations or domains from the Theoretical Domains Framework Version 2 [29] (a validated, comprehensive, theory-informed approach to identifying determinants of behaviour). Definition of domains referred to in this paper are provided in Box 1. Domain categorisations were confirmed by a second reviewer (DD).

Included studies were differentiated according to the study population, study design and whether factors were identified by participants *in response to an open question* or *endorsed from a list of factors presented by researchers*. In order to facilitate comparisons studies were grouped according to the summary statistics used and *p*-values and Odds Ratios compared where possible. We prioritised 1) the most ecologically valid data [30, 31] (i.e. real-life OHCA calls and accounts of people who had actually witnessed OHCA), 2) studies which formally assessed posited relationships and 3) methodologically strong studies (i.e. assessed as low risk of bias) in the findings section.

## Results

**Fig. 1:**
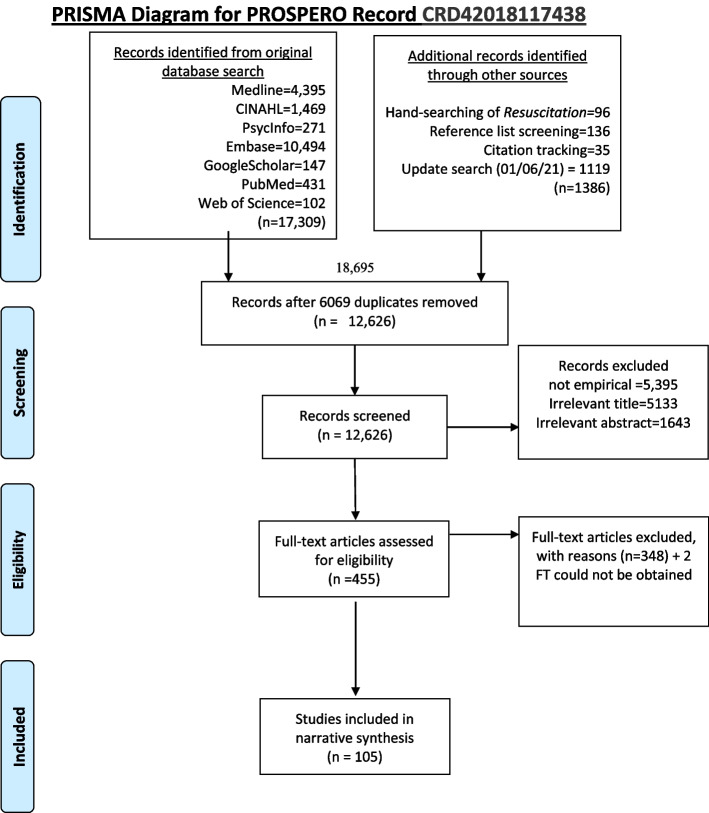
PRISMA diagram

Original database searches conducted on 13th Dec 2018 (see PRISMA diagram, Fig. 1) identified 17,309 citations with 87 studies included after screening for eligibility. An update search conducted 01/06/21 identified 1119 additional titles, 15 of which were assessed as eligible. Hand-searching of *Resuscitation* (Jan 2005-Dec 2021) identified 96 potentially relevant titles, seven of which had not already been identified by database screening, none met the inclusion criteria. Reference lists of included studies identified an additional 136 papers, 26 of which had not been previously identified, two studies were eligible and included. Finally, citation tracking identified 35 potentially relevant titles, seven not previously screened and one study included. Therefore, a total of 105 studies were included in the narrative synthesis.

### Description of included studies

Table 1 summarises the main characteristics of the 105 included studies comprising a total of 150,820 participants. The studies were published between 1989 and 2021 and conducted across 30 countries. The studies were heterogenous in design and included: randomised controlled trials (*n* = 6); non-randomised trials (*n* = 1); a quasi-experimental deign (*n* = 1), prospective cohort study (*n* = 1); before and after studies (*n* = 15); cross sectional studies (*n* = 67), qualitative studies (*n* = 9) and studies examining actual OHCA calls to Emergency Medical Services (*n* = 5).

**Table 1:** List of included studies

Author(s)	Country	Study Type	Participants	(n=)
Aaberg et al. 2014 [32]	Denmark	Before and after study	High School students	399
Alhussein et al. 2021 [33]	Saudi Arabia	Cross-sectional survey	Adults (≥18 years)	856
Alshudukhi et al. 2018 [34]	Saudi Arabia	Cross-sectional survey	Adults (≥18 years)	310
Anto-Ocra et al. 2020 [35]	Ghana	Cross-sectional survey	Adults (≥18 years)	277
Axelsson et al. 1996 [36]	Sweden	Cross-sectional survey	People who reported making a CPR attempt between 1990 and 1994	742
Axelsson et al. 2000 [37]	Sweden	Cross-sectional survey	Adults (≥18 years) who had received training in basic CPR in January 1997	1012
Babic et al. 2020 [38]	Slovenia	Cross-sectional survey	Adults (≥18 years)	198
Becker et al. 2019 [39]	USA	Cross-sectional survey	Adults (≥18 years) who attended CPR training event	677
Bin et al. 2013 [40]	Saudi Arabia	Cross-sectional survey	High school students	575
Birkun & Kosova 2018 [41]	Crimea	Cross-sectional survey	Adult (≥18 yrs)	384
Bohn et al. 2012 [42]	Germany	Prospective cohort	Grammar school pupils (age 10 and age 13)	280
Bouland et al. 2017 [43]	USA	Before and after study	Laypeople (≥14 years)	238
Bray et al. 2017 [44]	Australia	Cross-sectional survey	Adult (≥18 yrs)	404
Breckwoldt, Scholesser & Arntz 2009 [45]	Germany	Cross-sectional survey	Witnesses of an OHCA	138
Brinkrolf et al. 2018 [46]	Germany	Cross-sectional survey	Witnesses of an OHCA	101
Case et al. 2018 [47]	Australia	Retrospective analysis of emergency calls for OHCA	Calls to Dispatch Centre with OHCA	120
Chen et al. 2017 [48]	China	Cross-sectional survey	Adult laypersons (≥18 yrs) + 3 < 18 years	1841
Cheng et al. 1997 [49]	Taiwan	Cross-sectional survey	Families of cardiac patients and general public	856
Cheng-Yu et al. 2016 [50]	Taiwan	Before and after study	Adults (≥20 years)	401
Cheskes et al. 2016 [51]	Canada	Cross-sectional survey	Adult (≥18 yrs)	428
Chew et al. 2009 [52]	Malaysia	Cross-sectional survey	School teachers	73
Chew et al. 2019 [53]	Malaysia	Cross-sectional survey	Adult (min age NR) participants at a mass CPR training event	6248
Cho et al. 2010 [54]	Korea	Before and after study	Lay people aged 11 years and over	890
Compton et al. 2003 [55]	USA	Cross-sectional survey	School teachers	201
Coons & Guy 2009 [56]	USA	Cross-sectional survey	Adult (≥18 years)	755
Cu, Phan & O’Leary 2009 [57]	Australia	Cross-sectional survey	Caregivers of children presenting to the Emergency Department (≥18 years)	348
Dami et al. 2010 [58]	Switzerland	Retrospective analysis of emergency calls for OHCA	Call to Dispatch Centre with OHCA	738
De Smedt et al. 2018 [59]	Belgium	Cross-sectional survey	Schoolchildren aged 10–18, teachers and principals	929
Dobbie et al. 2018 [60]	Scotland	Cross-sectional survey	Adults (≥16 years)	1027
Donohoe, Haefeli & Moore 2006 [61]	England	Qualitative: focus groups	Adults (≥16 years)	NR
Dracup et al. 1994 [62]	USA	Randomised Controlled Trial	Family members of patients at risk of cardiac arrest	172
Dwyer 2008 [63]	Australia	Cross-sectional survey	Adults (≥18 years)	1208
Enami et al. 2010 [64]	Japan	Before and after study	Adults (≥17 years). New driver licence applicants	8890
Fratta et al. 2020 [65]	USA	Cross-sectional survey	Attendees at large public gatherings (aged ≥14)	516
Han et al. 2018 [66]	Korea	Before and after study	Family members (≥18 years) of patients at risk of cardiac arrest	203
Hauff et al. 2003 [67]	USA	Retrospective analysis of emergency calls for OHCA	Call to Dispatch Centre with OHCA	404
Hawkes et al. 2019 [68]	UK	Cross-sectional survey	Adults (≥18 years)	2084
Hollenberg et al. 2019 [69]	Sweden	Randomised Controlled Trial	School students (13 years)	641
Huang, Hu & Mao 2016 [70]	China	Cross-sectional survey	School and University students (13–21 years)	1407
Hubble et al. 2003 [71]	USA	Cross-sectional survey	High school students	683
Hung et al. 2017 [72]	Hong Kong	Cross-sectional survey	College and University students (≥15 years)	351
Iserbyt 2016 [73]	Belgium	Before and after study	Secondary school pupils	313
Jelinek et al. 2001 [74]	Australia	Cross-sectional survey	General public (age not recorded)	803
Johnston et al. 2003 [75]	Australia	Cross-sectional survey	Adults (≥18 years)	4490
Kandakai & King 1999 [76]	USA	Before and after study	College students	214
Kanstad, Nilsen & Fredriksen 2011 [77]	Norway	Cross-sectional survey	Secondary school students (16–19 years)	376
Karuthan et al. 2019 [78]	Malaysia	Cross-sectional survey	College students	393
Kua et al. 2018 [79]	Singapore	Before and after study	School students (11–17 years)	966
Kuramoto et al. 2008 [80]	Japan	Cross-sectional survey	Adults (≥15 years)	1132
Lam et al. 2007 [81]	Hong Kong	Cross-sectional survey	Laypersons who attended the CPR course (aged ≥7 years)	305
Lee et al. 2013 [82]	South Korea	Before and after study	College students	2029
Lerner et al. 2008 [83]	USA	Retrospective analysis of emergency calls for OHCA	Call to Dispatch Centre with OHCA	168
Lester, Donnelly & Weston 1997 [84]	Wales	Cross-sectional survey	First year high school pupils	233
Lester, Donnelly & Assar 1997 [85]	Wales	Cross-sectional survey	General public	241
Lester, Donnelly & Assar 2000 [86]	UK	Cross-sectional survey	Participants who had attended a CPR course	416
Liaw et al. 2020 [87]	Malaysia	Before and after study	University employees (non-medical)	184
Locke et al. 1995 [88]	USA	Cross-sectional survey	Lay people (minimum age not reported) & health care providers	975
Lu et al. 2017 [89]	China	Cross-sectional survey	College students	609
Lynch & Einspruch 2010 [90]	USA	Randomised Controlled Trial	Adults (≥18 years)	1065
Maes et al. 2015 [91]	Belgium	Before and after study	Hospital visitors (≥13 years)	85
Magid et al. 2019 [92]	USA	Cross-sectional survey	College students	588
Mathiesen et al. 2017 [93]	Norway	Qualitative: interviews	Witnesses of an OHCA	10
Mausz, Snobelen & Tavares 2018 [94]	Canada	Qualitative: interviews/focus groups	Witnesses of an OHCA	15
McCormack, Damon & Einsenberg 1989 [95]	USA	Cross-sectional survey	Witnesses of an OHCA	34
Mecrow et al. 2015 [96]	Bangladesh	Cross-sectional survey	Lay people (≥10 years)	721
Meischke et al. 2002 [97]	USA	Cross-sectional survey	Older adults (minimum age not reported)	159
Moller et al. 2014 [98]	Denmark	Qualitative: interviews	Witnesses of an OHCA	33
Nielsen et al. 2013 [99]	Denmark	Before and after study	Adults (≥15 years)	1639
Nishiyama et al. 2019 [100]	Japan	Cross-sectional survey	University students	5549
Nolan et al. 1999 [101]	Canada	Cross-sectional survey	Adults (≥45 years)	786
Nord et al. 2016 [102]	Sweden	Cluster randomised trial	Schoolchildren	1124
Nord et al. 2017 [103]	Sweden	Cluster randomised trial	Schoolchildren	587
Omi et al. 2008 [104]	Japan	Cross-sectional survey	High school students	3316
Onan et al. 2018 [105]	Turkey	Quasi-experimental study	High school students (aged 17–18)	77
Parnell et al. 2006 [106]	New Zealand	Cross-sectional survey	High school students	494
Pei-Chuan Huang et al. 2019 [107]	Taiwan	Cross-sectional survey	Adults (≥20 years)	1073
Platz et al. 2000 [108]	USA	Cross-sectional survey	Family members of patients at risk of cardiac arrest	100
Rankin et al. 2020 [109]	Australia	Cross-sectional survey	Adults (18–21 years)	178
Riou et al., 2020 [110]	Australia	Retrospective analysis of emergency calls for OHCA	Call to Ambulance service with OHCA where caller initially did not agree to perform CPR	65
Ro et al. 2016 [111]	Korea	Cross-sectional survey	Adults (≥19 years)	62,425
Rowe et al. 1998 [112]	Canada	Cross-sectional survey	Adults (≥44 years)	811
Sasaki et al. 2015 [113]	Japan	Cross-sectional survey	Adults (≥15 years)	4853
Sasson et al. 2013 [114]	USA	Qualitative: focus groups	Laypeople (minimum age not stated)	42
Sasson et al. 2015 [115]	USA	Qualitative: focus groups	Laypeople (≥13 years)	64
Schmid et al. 2016 [116]	Costa Rica	Cross-sectional survey	Laypeople (minimum age not stated)	370
Schmitz et al. 2015 [117]	Netherlands	Randomised Controlled Trial	High school students	201
Schneider et al. 2004 [118]	Austria	Before and after study	Survivors of OHCA and people who know them	112
Shams et al. 2016 [119]	Lebanon	Cross-sectional survey	University students	948
Shibata et al. 2000 [120]	Japan	Cross-sectional survey	High school students and teachers	626
Sipsma, Stubbs & Plorde 2011 [121]	USA	Cross-sectional survey	Adults (≥18 years)	1001
Skora & Riegel 2001 [122]	USA	Cross-sectional survey (qualitative analysis)	Laypersons who had provided out-of-hospital CPR to strangers	12
Smith et al. 2003 [123]	Australia	Cross-sectional survey	Householders (age not reported)	1489
Sneath & Lacey 2009 [124]	USA	Cross-sectional survey	Adults (≥18 years)	78
So et al. 2020 [125]	Hong Kong	Before and after study	High school students (12–15 years)	128
Swor et al. 2006 [20]	USA	Cross-sectional survey	Witnesses of an OHCA	684
Swor et al. 2013 [126]	USA	Cross-sectional survey	Witnesses of an OHCA	30
Tang et al. 2020 [127]	China	Cross-sectional survey	High school students (senior, age NR)	397
Taniguchi, Omi & Inaba 2007 [128]	Japan	Cross-sectional survey	High school students and teachers	3444
Taniguchi et al. 2012 [129]	Japan	Cross-sectional survey	High school students and teachers	1946
Thorén et al. 2010 [130]	Sweden	Qualitative: interviews	Partners of people who experienced OHCA	15
Vaillancourt et al. 2013 [131]	Canada	Cross-sectional survey	Adults (≥55 years)	192
Vetter et al. 2016 [132]	USA	Non-randomised trial	High school students	412
Wilks et al. 2015 [133]	Hong Kong	Cross-sectional survey	Secondary school students (15–16 years)	383
Winkelman et al. 2009 [134]	USA	Cross-sectional survey	Teacher candidates	582
Zinckernagel et al. 2016 [135]	Denmark	Qualitative: interviews and focus groups	Secondary school leaders and teachers	25

### Methodological quality

Of the quantitative studies, four [58, 69, 103, 110] were identified as strong, six as moderate [83, 90, 102, 117, 125, 131] with the remaining 87 quantitative studies rated ‘weak’ (see Table 2). There was a predominance of non-randomised designs, uncontrolled confounders, and use of unvalidated data collection methods. All qualitative studies were assessed as of sufficient quality for inclusion but also varied in quality (*n* = 8).

**Table 2:** EPHPP Quality Assessment of included studies

	Selection Bias	Study Design	Confounders	Blinding	Data Collection Method	Withdrawals & Drop outs	Global rating
Aaberg et al. 2014 [32]	Moderate	Moderate	Strong	Weak	Weak	Moderate	Weak
Alhussein et al. 2021 [33]	Moderate	Weak	Strong	Weak	Strong	Moderate	Weak
Alshudukhi et al. 2018 [34]	Weak	Weak	Weak	Weak	Weak	Moderate	Weak
Anto-Ocra et al. [35]	Moderate	Weak	Strong	Weak	Moderate	Moderate	Weak
Axelsson et al. 1996 [36]	Moderate	Weak	Weak	Weak	Weak	Moderate	Weak
Axelsson et al. 2000 [37]	Moderate	Weak	Weak	Weak	Moderate	Moderate	Weak
Babic et al. 2020 [38]	Weak	Weak	Weak	Weak	Weak	Moderate	Weak
Becker et al. 2019 [39]	Moderate	Weak	Weak	Weak	Weak	Moderate	Weak
Bin et al. 2013 [40]	Weak	Weak	Weak	Weak	Weak	Moderate	Weak
Birkun & Kosova 2018 [41]	Moderate	Weak	Weak	Weak	Strong	Moderate	Weak
Bohn et al. 2012 [42]	Moderate	Moderate	Weak	Weak	Weak	Weak	Weak
Bouland et al. 2017 [43]	Moderate	Weak	Weak	Weak	Moderate	Strong	Weak
Bray et al. 2017 [44]	Moderate	Weak	Weak	Weak	Weak	Moderate	Weak
Breckwoldt, Scholesser & Arntz 2009 [45]	Moderate	Weak	Weak	Weak	Weak	Moderate	Weak
Brinkrolf et al. 2018 [46]	Weak	Weak	Weak	Weak	Weak	Weak	Weak
Case et al. 2018 [47]	Moderate	Weak	Weak	Moderate	Strong	Moderate	Weak
Chen et al. 2017 [48]	Weak	Weak	Weak	Weak	Weak	Strong	Weak
Cheng et al. 1997 [49]	Weak	Weak	Moderate	Weak	Weak	Strong	Weak
Cheng-Yu et al. 2016 [50]	Moderate	Weak	Weak	Weak	Weak	Weak	Weak
Cheskes et al. 2016 [51]	Moderate	Weak	Weak	Weak	Strong	Moderate	Weak
Chew et al. 2009 [52]	Weak	Weak	Weak	Weak	Weak	Moderate	Weak
Chew et al. 2019 [53]	Weak	Weak	Moderate	Weak	Strong	Weak	Weak
Cho et al. 2010 [54]	Moderate	Moderate	Weak	Weak	Weak	Moderate	Weak
Compton et al. 2003 [55]	Strong	Weak	Weak	Weak	Weak	Moderate	Weak
Coons & Guy 2009 [56]	Moderate	Weak	Weak	Moderate	Strong	Weak	Weak
Cu, Phan & O’Leary 2009 [57]	Moderate	Weak	Weak	Weak	Strong	Weak	Weak
Dami et al. 2010 [58]	Strong	Moderate	Moderate	Moderate	Strong	Moderate	Strong
De Smedt et al. 2018 [59]	Weak	Weak	Weak	Weak	Moderate	Moderate	Weak
Dobbie et al. 2018 [60]	Moderate	Weak	Weak	Weak	Strong	Weak	Weak
Dracup et al. 1994 [62]	Moderate	Moderate	Weak	Moderate	Weak	Moderate	Weak
Dwyer 2008 [63]	Moderate	Weak	Weak	Weak	Moderate	Weak	Weak
Enami et al. 2010 [64]	Weak	Weak	Weak	Moderate	Weak	Weak	Weak
Fratta et al. 2020 [65]	Moderate	Weak	Moderate	Weak	Weak	Moderate	Weak
Han et al. 2018 [66]	Moderate	Weak	Weak	Weak	Weak	Weak	Weak
Hauff et al. 2003 [67]	Weak	Weak	Weak	Moderate	Strong	Moderate	Weak
Hawkes et al. 2019 [68]	Moderate	Weak	Weak	Weak	Weak	Moderate	Weak
Hollenberg et al. 2019 [69]	Moderate	Strong	Strong	Moderate	Strong	Strong	Strong
Huang, Hu & Mao 2016 [70]	Weak	Weak	Weak	Weak	Moderate	Moderate	Weak
Hubble et al. 2003 [71]	Weak	Weak	Weak	Weak	Weak	Weak	Weak
Hung et al. 2017 [72]	Weak	Weak	Weak	Weak	Strong	Strong	Weak
Iserbyt 2016 [73]	Weak	Weak	Weak	Weak	Moderate	Strong	Weak
Jelinek et al. 2001 [74]	Weak	Weak	Weak	Weak	Weak	Weak	Weak
Johnston et al. 2003 [75]	Moderate	Weak	Weak	Moderate	Strong	Weak	Weak
Kandakai & King 1999 [76]	Weak	Weak	Weak	Weak	Strong	Weak	Weak
Kanstad, Nilsen & Fredriksen 2011 [77]	Weak	Weak	Weak	Moderate	Weak	Weak	Weak
Karuthan et al. 2019 [78]	Moderate	Weak	Moderate	Weak	Weak	Moderate	Weak
Kua et al. 2018 [79]	Weak	Weak	Weak	Moderate	Weak	Moderate	Weak
Kuramoto et al. 2008 [80]	Weak	Weak	Weak	Weak	Moderate	Weak	Weak
Lam et al. 2007 [81]	Weak	Weak	Weak	Weak	Weak	Moderate	Weak
Lee et al. 2013 [82]	Moderate	Weak	Weak	Weak	Moderate	Weak	Weak
Lerner et al. 2008 [83]	Strong	Moderate	Moderate	Moderate	Weak	Moderate	Moderate
Lester, Donnelly & Weston 1997 [84]	Weak	Weak	Weak	Weak	Weak	Strong	Weak
Lester, Donnelly & Assar 1997 [85]	Weak	Weak	Weak	Weak	Weak	Weak	Weak
Lester, Donnelly & Assar 2000 [86]	Weak	Weak	Weak	Weak	Weak	Weak	Weak
Liaw et al. 2020 [87]	Weak	Weak	Moderate	Weak	Weak	Moderate	Weak
Locke et al. 1995 [88]	Weak	Weak	Weak	Weak	Weak	Weak	Weak
Lu et al. 2016 [89]	Weak	Weak	Weak	Weak	Moderate	Strong	Weak
Lynch & Einspruch 2010 [90]	Moderate	Moderate	Weak	Moderate	Moderate	Moderate	Moderate
Maes et al. 2015 [91]	Weak	Weak	Weak	Weak	Weak	Weak	Weak
Magid et al. 2019 [92]	Weak	Weak	Weak	Weak	Moderate	Strong	Weak
Mecrow et al. 2015 [96]	Weak	Weak	Weak	Weak	Weak	Weak	Weak
Meischke et al. 2002 [97]	Weak	Weak	Weak	Weak	Weak	Weak	Weak
Nielsen et al. 2013 [99]	Moderate	Weak	Moderate	Moderate	Weak	Moderate	Weak
Nishiyama et al. 2019 [100]	Moderate	Weak	Moderate	Weak	Weak	Moderate	Weak
Nolan et al. 1999 [101]	Moderate	Strong	Weak	Moderate	Weak	Weak	Weak
Nord et al. 2016 [102]	Strong	Strong	Weak	Strong	Strong	Moderate	Moderate
Nord et al. 2017 [103]	Moderate	Moderate	Moderate	Moderate	Moderate	Strong	Strong
Omi et al. 2008 [104]	Moderate	Weak	Weak	Weak	Weak	Strong	Weak
Onan et al. 2018 [105]	Weak	Weak	Weak	Weak	Weak	Strong	Weak
Parnell et al. 2006 [106]	Weak	Weak	Weak	Weak	Weak	Weak	Weak
Pei-Chuan Huang et al. 2019 [107]	Moderate	Moderate	Weak	Weak	Strong	Weak	Weak
Platz et al. 2000 [108]	Weak	Weak	Weak	Weak	Moderate	Strong	Weak
Rankin et al. 2020 [109]	Weak	Weak	Moderate	Weak	Weak	Moderate	Weak
Riou et al. 2020 [110]	Strong	Moderate	Moderate	Moderate	Strong	Strong	Strong
Ro et al. 2016 [111]	Moderate	Weak	Weak	Weak	Strong	Weak	Weak
Rowe et al. 1998 [112]	Moderate	Weak	Weak	Weak	Moderate	Weak	Weak
Sasaki et al. 2015 [113]	Moderate	Weak	Weak	Moderate	Weak	Weak	Weak
Schmid et al. 2016 [116]	Weak	Weak	Weak	Weak	Moderate	Strong	Weak
Schmitz et al. 2015 [117]	Moderate	Strong	Weak	Moderate	Moderate	Moderate	Moderate
Schneider et al. 2004 [118]	Weak	Weak	Weak	Weak	Weak	Weak	Weak
Shams et al. 2016 [119]	Moderate	Weak	Weak	Weak	Weak	Moderate	Weak
Shibata et al. 2000 [120]	Weak	Weak	Weak	Weak	Weak	Moderate	Weak
Sipsma, Stubbs & Plorde 2011 [121]	Weak	Weak	Weak	Weak	Weak	Weak	Weak
Skora & Riegel 2001 [122]	Weak	Weak	Weak	Moderate	Weak	Moderate	Weak
Smith et al. 2003 [123]	Weak	Weak	Weak	Weak	Weak	Weak	Weak
Sneath & Lacey 2009 [124]	Weak	Weak	Weak	Weak	Weak	Weak	Weak
So et al. 2020 [125]	Moderate	Moderate	Moderate	Weak	Moderate	Strong	Moderate
Swor et al. 2006 [20]	Moderate	Moderate	Weak	Weak	Weak	Moderate	Weak
Swor et al. 2013 [126]	Weak	Weak	Weak	Moderate	Weak	Moderate	Weak
Tang et al. 2020 [127]	Moderate	Weak	Moderate	Weak	Weak	Moderate	Weak
Taniguchi, Omi & Inaba 2007 [128]	Weak	Weak	Weak	Weak	Weak	Moderate	Weak
Taniguchi et al. 2012 [129]	Weak	Weak	Weak	Weak	Weak	Moderate	Weak
Vaillancourt et al. 2013 [131]	Moderate	Moderate	Weak	Strong	Moderate	Moderate	Moderate
Vetter et al. 2016 [132]	Weak	Weak	Weak	Weak	Weak	Weak	Weak
Wilks et al. 2015 [133]	Moderate	Weak	Moderate	Weak	Moderate	Moderate	Weak
Winkelman et al. 2009 [134]	Weak	Weak	Weak	Weak	Moderate	Moderate	Weak
Zinckernagel et al. 2016 [135]	Weak	Weak	Weak	Weak	Moderate	Strong	Weak

**Fig. 2:**
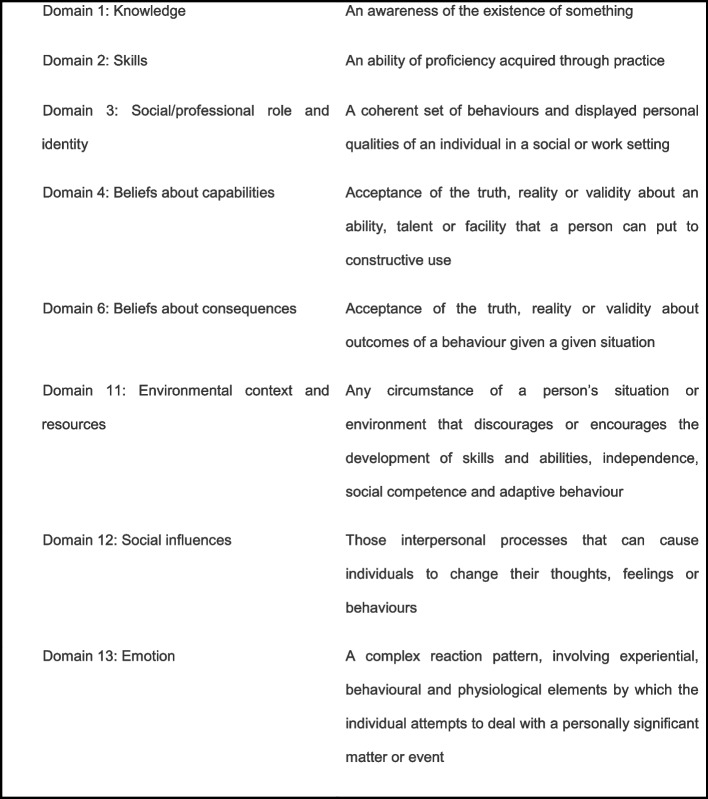
Theoretical Domains Framework definitions [29]

The psychological and behavioural factors identified from the included studies are reported below and summarised in Tables 3, 4, 5, 6, 7, 8, 9 and 10 below. Studies were divided into subgroups according to the study population (i.e. results from those with direct experience versus general samples responding to a ‘hypothetical’ OHCA); study design and statistics used. Data were further categorised depending on whether the ‘predictor’ was identified by participants in response to an open question or whether it was presented as a possible factor and subsequently endorsed. Factors are presented in relation to the domains of the Theoretical Domains Framework so that theoretically similar factors are grouped together and can be compared across study designs (Fig. 2).

**Table 3:** Psychological and behavioural factors associated with LOWER actual/intended CPR initiation (grouped using Theoretical Domains Framework V.2 [29])

Factor related to reluctance		Participants	Number (total)	Number in analysis for each factor	Unprompted identification of each factor (% of whole sample and % of *unwilling subsample*)	Endorsement of each factor when prompted (% of whole sample and % of *unwilling subsample*)
**6. Beliefs about Consequences**						
***Concerns about doing something wrong***						
Aaberg et al. 2014 [32]		High School students	399	399 responding as to their worst fear	NR (1 of 3 qualitative themes identified)	
Compton et al. 2003 [55]		School teachers	201	180		71% of untrained
						42% of trained
Coons & Guy 2009 [56]		Adults (≥18)	755	435 (who endorsed reasons)		20% (stranger)
						22.5% (family)
Dwyer 2008 [56]		Adults (≥18)	1208	379 (not confident)		*55%*
Iserbyt 2016 [63]		Secondary school pupils	313	313		38% (girls)
						26% (boys)
Nolan et al. 1999 [101]		Adults (≥45)	786	203 (not ready to perform CPR)		*53%*
Onan et al. 2018 [105]		High School students	83	83		NR (concern identified)
Sasson et al. 2013 [114]		Lay-people (min age not stated)	42	42	NR (1 of 10 qualitative barriers)	
Swor 2006 [20]		Witnesses of OHCA	684	279 (did not perform CPR)		*11%*
Tang et al. 2020 [127]		High school students (senior, age NR)	397	397		78% (fail to meet professional standards)
Zinckernagel et al. 2016 [135]		Secondary school leaders and teachers	25	25	NR (a qualitative theme identified)	
***Concerns about doing harm***						
Aaberg et al. 2014 [32]		High School students	399	399 responding as to their worst fear	NR (1 of 3 qualitative themes identified)	
Alhussein 2021 [33]		Adults (≥18)	856	Those whose source of knowledge was media sources (largest group) (*n* = 331)		Break rib 22% (family/friend)
						Break rib 21% (stranger)
						Organ damage 14% (family/friend)
						Organ damage 12% (stranger)
						Stopping heart 8% (family/friend)
						Stopping heart 5% (stranger)
Anto-Ocra et al. 2020 [35]		Adults (≥18 years)	277	277		35%
Babic et al. 2020 [38]		Adults (≥18 years)	198	198		15% (MMV)
						23% (compressions)
Becker et al. 2019 [39]		Adults (≥18 years) who attended CPR training event	677	306 resp. concerns elderly patient	63%	
				249 resp. concerns for woman	21%	
				291 resp. concerns for child	51%	
Cheng-Yu et al. 2016 [50]		Adults (≥20)	401	144 (unwilling to perform on stranger)		11%
Compton et al. 2003 [55]		School teachers	201	180		64% of untrained
						41% of trained
Coons & Guy 2009 [56]		Adults (≥18)	755	435 (who endorsed reasons)		19.4% stranger
						26.4% (family)
Cu 2009 [50]		Caregivers of children presenting to the Emergency Department (≥18 years)	348	125 (unwilling to perform CPR on adult)		*38%*
Dami 2010 [51]		Call to Dispatch Centre with OHCA	738	73 medically appropriate who refused	*3%*	
Dobbie 2018 [53]		Adults (≥16 years)	1027	1027		22%
Donohoe 2006 [54]		Adults (≥16 years)	NR	Focus groups (NR)	NR (Qualitative theme identified)	
Dwyer 2008 [56]		Adults (≥18)	1208	379 (not confident)		*10%*
Han 2018 [58]		Family members (≥18 years) of patients at risk of cardiac arrest	203	88		7%
Huang 2016 [60]		School and University students (13–21 years)	1407	546 (unwilling to perform on stranger)		*68%*
Hubble 2003 [61]		High school students	683	683	*25% (MMV)*	
					*31% (AED)*	
					*25% (CC)*	
Hung 2017 [62]		College and University students (≥15 years)	351	351		26%
Kandakai & King 1999 [76]		College students	214	214		65%
Kanstad, Nilsen & Fredriksen 2011 [77]		Secondary school students (16–19 years)	376	376		17%
Karuthan et al. 2019 [78]		College students	393	393		5% (HO stranger)
						3% (HO family-member)
Kua et al. 2018 [79]		School students (11–17 years)	1196	966		58%
Liaw et al. 2020 [87]		University employees (non-medical)	184	NR	Fear and concern identified as significantly reduced by training in 54%	
Maes et al. 2015 [91]^a^		Hospital visitors (≥13 years)	85	51 who did not feel able to use AED	*2%*	
Omi 2008 [91]		High school students	3316	2203 unwilling to perform CPR	*23%*	
Onan et al. 2018 [105]		High School students	83	83		NR (concern identified)
Magid et al. 2019 [92]		College students	588	300 (who identified barriers)		*52%*
Pei-Chuan Huang et al. 2019 [107]		Adults (≥20)	1073	141 (who provided reasons why not)		*36.5%*
Platz et al. 2000 [108]		Family members of patients at risk of cardiac arrest	100	100		49%
Rankin et al. 2020 [109]		Adults (18–21 years)	178			Not CPR trained, for family 76%
						CPR trained, for family 67%
						Not CPR trained, for stranger 69%
						CPR trained, for stranger 57%
Sasson et al. 2013 [114]		Lay-people (min age not stated)	42	42	NR (1 of 10 qualitative barriers)	
Schmid et al. 2016 [116]		Laypeople (minimum age not stated)	370	370		17.30%
Shams et al. 2016 [119]		University students	948	948		53%
So et al. 2020 [125]		High school students (12–15 years)	128	NR		94%
Swor 2006 [20]		Witnesses of OHCA	684	279 (did not perform CPR)		*2%*
Taniguchi 2012 [112]		High school students and teachers	1946	1708 (students on a stranger)		*14%*
Thoren 2010 [113]		Partners of people who experienced OHCA	15	15	NR (Qualitative theme identified)	
Wilks et al. 2015 [133]		Secondary school students (15–16 years)	383	NR		*28%*
***Concern about being the cause of the person’s death***						
Aaberg et al. 2014 [32]		High School students	399	399 responding as to their worst fear	NR (1 of 3 qualitative themes identified)	
Becker et al. 2019 [39]		Adults (≥18 years) who attended CPR training event	677	306 resp. concerns elderly patient	6%	
				249 resp. concerns for woman	2%	
				291 resp. concerns for child	4%	
Nolan et al. 1999 [101]		Adults (≥45)	786	203 (not ready to perform CPR)		*40%*
Onan et al. 2018 [105]		High School students	83	83		NR (concern identified)
Tang et al. 2020 [127]		High school students (senior, age NR)	397	397		9% (fear of treating dying person)
***Belief CPR futile***						
Axelsson et al. 1996 [36]		People who reported making a CPR attempt between 1990 and 1994	742	51 bystanders described hesitation	*NR*	
Case et al. 2018 [47]		OHCA Calls	120	120 calls where no CPR given	*28%*	
Hauff 2003 [59]		Call to Dispatch Centre with OHCA	404	52 who did not accept CPR instructions	*23%*	
Nolan et al. 1999 [101]		Adults (≥45)	786	203 (not ready to perform CPR)		*34%*
Riou et al. 2020 [110]		Retrospective analysis of emergency calls for OHCA	65	57 (where caller responded with an account)	50% expressed an ‘epistemic’ account – i.e. too late or futile	
Skora & Riegel 2001 [122]		Previously performed CPR	12	12 participants	NR (Qualitative theme identified)	
Swor 2006 [20]		Witnesses of OHCA	684	279 (did not perform CPR)		*4%*
***Belief CPR does not work***						
Babic et al. 2020 [38]		Adults (≥18 years)	198	198		0.5% (MMV)
						1% (compressions)
Schmid et al. 2016 [116]		Laypeople (minimum age not stated)	370	370		10%
Shams et al. 2016 [119]		University students	948	948		9%
***Violates beliefs about death***						
Hubble 2003 [61]		High school students	683	683	*4% (MMV)*	
					*4% (AED)*	
					*4% (CC)*	
Schmid et al. 2016 [116]		Laypeople (minimum age not stated)	370	370		5%
***Concerns about MMV***						
Anto-Ocra et al. 2020 [35]		Adults (≥18 years)	277	277		5%
Axelsson et al. 1996 [36]		People who reported making a CPR attempt between 1990 and 1994	742	51 bystanders described hesitation	*NR*	
Cheng-Yu et al. 2016 [50]		Adults (≥20)	401	144 (unwilling to perform on stranger)		*46%*
Cho et al. 2010 [54]		Lay people aged 11 years and over	890	539 (unwilling to perform CPR)		*17%*
Coons & Guy 2009 [56]		Adults (≥18)	755	435 (who endorsed reasons)		19% (stranger)
						16.5% (family)
Dobbie 2018 [53]		Adults (≥16 years)	1027	1027		7%
Donohoe 2006 [54]		Adults (≥16 years)	NR	Focus groups (NR)	NR (Qualitative theme identified)	
Iserbyt 2016 [63]		Secondary school pupils	313	313		19% (girls)
						10% (boys)
Pei-Chuan Huang et al. 2019 [107]		Adults (≥20)	1073	141 (who provided reasons why not)		*13%*
Sasson et al. 2013 [114]		Lay-people (min age not stated)	42	42	NR (1 of 10 qualitative barriers identified)	
Schmid et al. 2016 [116]		Laypeople (minimum age not stated)	370	370		18%
Shams et al. 2016 [119]		University students	948	948		19%
Swor 2006 [20]		Witnesses of OHCA	684	279 (did not perform CPR)		*1%*
***Concern about legal ramifications***						
Alhussein 2021 [33]		Adults (≥18)	856	Those whose source of knowledge was media sources (largest group) (*n* = 331)		5% (family/friend)
						22% (stranger)
Alshudukhi et al. 2018 [34]		Adults (≥18)	310	168 unwilling to perform CPR		*2%*
Anto-Ocra et al. 2020 [35]		Adults (≥18 years)	277	277		8%
Chen et al. 2017 [48]		Adult laypersons (≥18 yrs) + 3 < 18 years	1841	1841		53%
Cheng-Yu et al. 2016 [50]		Adults (≥20)	401	144 (unwilling to perform on stranger)		*37%*
Cho et al. 2010 [54]		Lay people aged 11 years and over	890	539 (unwilling to perform CPR)		*55%*
Compton et al. 2003 [55]		School teachers	201	180		52% of untrained
						54% of trained
Coons & Guy 2009 [56]		Adults (≥18)	755	435 (who endorsed reasons)		20.9% (stranger)
						12.1% (family)
Dobbie 2018 [53]		Adults (≥16 years)	1027	1027		8%
Donohoe 2006 [54]		Adults (≥16 years)	NR	Focus groups (NR)	NR (Qualitative theme identified)	
Huang 2016 [60]		School and University students (13–21 years)	1407	546 (unwilling to perform on stranger)		*91%*
Hubble 2003 [61]		High school students	683	NR	*16% (MMV)*	
					*17% (AED)*	
					*13% (CC)*	
Hung 2017 [62]		College and University students (≥15 years)	351	351		17%
Iserbyt 2016 [63]		Secondary school pupils	313	313		4% (girls)
						6% (boys)
Jelinek 2001 [64]		General public (age not reported)	803	84 unwilling to perform MMV	*4%*	
				26 unwilling to perform CC	*19%*	
Johnston 2003 [65]		Adults (≥18 years)	4490	4490	2%	
Kandakai & King 1999 [76]		College students	214	214		48%
Karuthan et al. 2019 [78]		College students	393	393		1% (HO stranger)
						1% (HO family-member)
Lerner et al. 2008 [83]		Call to Dispatch Centre with OHCA	168	145 who did not follow CPR instructions	*1%*	
Liaw et al. 2020 [87]		University employees (non-medical)	184	NR	Fear and concern identified as significantly reduced by training in 59%	
Lu et al. 2016 [89]		College students	609	609 (non-medical)		7–21% (dep on subject)
Nolan et al. 1999 [101]		Adults (≥45)	786	203 (not ready to perform CPR)		*38%*
Pei-Chuan Huang et al. 2019 [107]		Adults (≥20)	1073	141 (who provided reasons why not)		*44%*
Rankin et al. 2020 [109]		Adults (18–21 years)	178			Not CPR trained, for family 32%
						CPR trained, for family 26%
						Not CPR trained, for stranger 60%
						CPR trained, for stranger 75%
Sasson et al. 2013 [114]		Lay-people (min age not stated)	42	42	NR (1 of 10 qualitative barriers identified)	
Sasson et al. 2015 [115]		Lay-people (≥13)	64	64	NR (qualitative barrier identified)	
Schmid et al. 2016 [116]		Laypeople (minimum age not stated)	370	370		30%
Shams et al. 2016 [119]		University students	948	948		25%
So et al. 2020 [125]		High school students (12–15 years)	128	NR		52%
Tang et al. 2020 [127]		High school students (senior, age NR)	397	397		67%
Wilks et al. 2015 [133]		Secondary school students (15–16 years)	383	NR		*14%*
Winkelman et al. 2009 [134]		Teacher candidates	582	47		*17%*
***Concerns about risk to self***						
Becker et al. 2019 [39]		Adults (≥18 years) who attended CPR training event	677	306 resp. concerns elderly patient	2%	
				249 resp. concerns for woman	2%	
				291 resp. concerns for child	1%	
Cu 2009 [50]		Caregivers of children presenting to the Emergency Department (≥18 years)	348	125 (unwilling to perform CPR on adult)		*3%*
Hubble 2003 [61]		High school students	683	NR	*7% (MMV)*	
					*10% (AED)*	
					*6% (CC)*	
Jelinek 2001 [64]		General public (age not reported)	803	84 unwilling to perform MMV	*56%*	
Johnston 2003 [65]		Adults (≥18 years)	4490	4490	4.50%	
Lester, Donnelly & Weston 1997 [84]		First year high school pupils	233	233	11%	
Liaw et al. 2020 [87]		University employees (non-medical)	184	NR	Fear and concern identified as significantly reduced by training in 34%	
Mathiesen et al. 2017 [93]		Witnesses of OHCA	10	10	NR (qualitative barrier identified)	
Sasson et al. 2013 [114]		Lay-people (min age not stated)	42	42	NR (1 of 10 qualitative barriers)	
Sasson et al. 2015 [115]		Lay-people (≥13)	64	64	NR (qualitative barrier identified)	
***Concerns about risk of infection***						
Alhussein 2021 [33]		Adults (≥18)	856	Those whose source of knowledge was media sources (largest group) (*n* = 331)		2% (family/friend)
						5% (stranger)
Anto-Ocra et al. 2020 [35]		Adults (≥18 years)	277	277		18%
Axelsson et al. 2000 [37]		Adults (≥18 years) who had received training in basic CPR in January 1997.	1012	1012		8%
Babic et al. 2020 [38]		Adults (≥18 years)	198	198		15% (MMV)
						0.5% (compressions)
Chen et al. 2017 [48]		Adult laypersons (≥18 yrs) + 3 < 18 years	1841	1841		2%
Cheskes et al. 2016 [51]		Adult (≥18 yrs)	428	NR	*24%*	
Cho et al. 2010 [54]		Lay people aged 11 years and over	890	539 (unwilling to perform CPR)		*10%*
Compton et al. 2003 [55]		School teachers	201	180		50% of untrained
						58% of trained
Dami 2010 [51]		Call to Dispatch Centre with OHCA	738	73 medically appropriate who refused	*4%*	
Dobbie 2018 [53]		Adults (≥16 years)	1027	1027		10%
Donohoe 2006 [54]		Adults (≥16 years)	NR	Focus groups (n NR)	NR (Qualitative theme identified)	
Dwyer 2008 [56]		Adults (≥18)	1208	379 (not confident)		*1%*
Han 2018 [58]		Family members (≥18 years) of patients at risk of cardiac arrest	203	88		1%
Huang 2016 [60]		School and University students (13–21 years)	1407	546 (unwilling to perform on stranger)		*24%*
Hubble 2003 [61]		High school students	683	NR	*35% (MMV)*	
					*11% (AED)*	
					*12% (CC)*	
Hung 2017 [62]		College and University students (≥15 years)	351	351		8%
Iserbyt 2016 [63]		Secondary school pupils	313	313		10% (girls)
						11% (boys)
Jelinek 2001 [64]		General public (age not reported)	803	84 unwilling to perform MMV	*19% (MMV)*	
Johnston 2003 [65]		Adults (≥18 years)	4490	4490	18%	
Kanstad, Nilsen & Fredriksen 2011 [77]		Secondary school students (16–19 years)	376	376		6%
Karuthan et al. 2019 [78]		College students	393	393		3% (HO stranger)
						1% (HO family-member)
Lee et al. 2013 [82]		College students	2029	242 (unwilling to perform CPR)		*< 1%*
Lester, Donnelly & Weston 1997 [84]		First year high school pupils	233	233	12% (7% HIV, 5% other)	
Lester, Donnelly & Assar 1997 [85]		General public	241	241	8% (5% HIV, 3% other)	
Liaw et al. 2020 [87]		University employees (non-medical)	184	NR	Fear and concern identified as significantly reduced by training in 34%	
Lu et al. 2016 [89]		College students	609	609 (non-medical)		10–45% (dep on subject)
Nolan et al. 1999 [101]		Adults (≥45)	786	203 (not ready to perform CPR)		*36%*
Omi 2008 [91]		High school students	3316	2203 unwilling to perform CPR	*11% (of 2203 who were unwilling)*	
Pei-Chuan Huang et al. 2019 [107]		Adults (≥20)	1073	141 (who provided reasons why not)		*28%*
Platz et al. 2000 [108]		Family members of patients at risk of cardiac arrest	100	100		9%
Rankin et al. 2020 [109]		Adults (18–21 years)	178			Not CPR trained, for family 6%
						CPR trained, for family 15%
						Not CPR trained, for stranger 32%
						CPR trained, for stranger 44%
Shams et al. 2016 [119]		University students	948	948		33%
Shibata 2000 [105]		Schoolchildren and teachers	626	NR		*5%*
So et al. 2020 [125]		High school students (12–15 years)	128	NR		28%
Skora & Riegel 2001 [122]		Previously performed CPR	12	12 participants	*8%*	
Tang et al. 2020 [127]		High school students (senior, age NR)	397	397		23%
Taniguchi 2007 [111]		High school students and teachers	3444	3444		*10%*
Taniguchi 2012 [112]		High school students and teachers	1946	1708 students on a stranger		*7%*
Wilks et al. 2015 [133]		Secondary school students (15–16 years)	383	NR		*6%*
Winkelman et al. 2009 [134]		Teacher candidates	582	47		*30%*
***Delaying CPR won’t do harm***						
Magid et al. 2019 [92]		College students	588	300 (who identified barriers)		*3.5%*
***Concerns about substance use***						
***Drugs***						
Dobbie 2018 [53]		Adult (> 16)	1027	1027		16%
Johnston 2003 [65]		Adults (≥18 years)	4490	4490	2%	
***Alcohol***						
Dobbie 2018 [53]		Adults (≥16 years)	1027	1027		
Johnston 2003 [65]		Adults (≥18 years)	4490	4490	2%	10%
**4. Beliefs about capabilities**						
***Concerns about capability (general)***						
Alhussein 2021 [33]		Adults (≥18)	856	Those whose source of knowledge was media sources (largest group) (*n* = 331)		84% (family/friend)
						83% (stranger)
Alshudukhi et al. 2018 [34]		Adults (≥18)	310	168 unwilling to perform CPR		61%
Anto-Ocra et al. 2020 [35]		Adults (≥18 years)	277	277		61%
Babic et al. 2020 [38]		Adults (≥18 years)	198	198		37% (MMV)
						32% (compressions)
Becker et al. 2019 [39]		Adults (≥18 years) who attended CPR training event	677	306 resp. concerns elderly patient	13%	
				249 resp. concerns for woman	14%	
				291 resp. concerns for child	23%	
Chen et al. 2017 [48]		Adult laypersons (≥18 yrs) + 3 < 18 years	1841	1841		44%
Cheng-Yu et al. 2016 [50]		Adults (≥20)	401	144 (unwilling to perform on stranger)		*6%*
Cho et al. 2010 [54]		Lay people aged 11 years and over	890	539 (unwilling to perform CPR)		*50%*
Cu 2009 [50]		Caregivers of children presenting to the Emergency Department (≥18 years)	348	125 (unwilling to perform CPR on adult)		*77%*
Dobbie 2018 [53]		Adults (≥16 years)	1027	1027		19%
Huang 2016 [60]		School and University students (13–21 years)	1407	546 (unwilling to perform on stranger)		*53%*
Iserbyt 2016 [63]		Secondary school pupils	313	313		31% (girls)
						23% (boys)
Johnston 2003 [65]		Adults (≥18 years)	4490	4490	2%	
Karuthan et al. 2019 [78]		College students	393	393		36% (HO stranger)
						27% (HO family-member)
Kanstad, Nilsen & Fredriksen 2011 [77]		Secondary school students (16–19 years)	376	376		79%
Maes et al. 2015 [91]^a^		Hospital visitors (≥13 years)	85	51 who did not feel able to use AED	45% (Don’t know how AED works)	
Nielsen et al. 2013 [99]		Adults (≥15 years)	1639	*n* = 114 (unwilling to provide CC, 2008)		*54%*
				*n* = 94 (unwilling to provide MMV, 2008)		*44%*
				*n* = 89 (unwilling to provide CC, 2009)		*48%*
				*n* = 90 (unwilling to provide MMV, 2009)		*35%*
Omi 2008 [91]		High school students	3316	2203 unwilling to perform CPR	*55% (of 2203 who were unwilling)*	
Pei-Chuan Huang et al. 2019 [107]		Adults (≥20)	1073	141 (who provided reasons why not)		*12%*
Platz et al. 2000 [108]		Family members of patients at risk of cardiac arrest	100	100		35%
Rankin et al. 2020 [109]		Adults (18–21 years)	178			Not CPR trained, for family 65%
						CPR trained, for family 68%
						Not CPR trained, for stranger 58%
						CPR trained, for stranger 57%
Shams et al. 2016 [119]		University students	948	948		56%
Shibata 2000 [105]		Schoolchildren and teachers	626	NR		*80%*
Sipsma, Stubbs & Plorde 2011 [121]		Adults (≥18)	1001	333		*33%*
Taniguchi 2007 [111]		High school students and teachers	3444	3444		*70%*
Taniguchi 2012 [112]		High school students and teachers	1946	1708 students on a stranger		*67%*
Winkelman et al. 2009 [134]		Teacher candidates	582	47		*38%*
***Concerns about physical capability***						
Case et al. 2018 [47]		OHCA Calls	120	120 calls where no CPR given	*15%*	
Coons & Guy 2009 [56]		Adults (≥18)	755	435 (who endorsed reasons)		21.5% (stranger)
						22.5% (family)
Dami 2010 [51]		High school students	3316	2203 unwilling to perform CPR	*55% (of 2203 who were unwilling)*	
Hauff 2003 [59]		Call to Dispatch Centre with OHCA	404	52 who did not accept CPR instructions	*11%*	
Jelinek 2001 [64]		General public (age not reported)	803	26 unwilling to perform CC	*11%*	
Lerner et al. 2008 [83]		Call to Dispatch Centre with OHCA	168	145 who did not follow CPR instructions	*8%*	
Lu et al. 2016 [89]		College students	609	609 (non-medical)		1–3% (dep on subject)
Pei-Chuan Huang et al. 2019 [107]		Adults (≥20)	1073	141 (who provided reasons why not)		*1.3%*
Platz et al. 2000 [108]		Family members of patients at risk of cardiac arrest	100	100		14%
Riou et al. 2020 [110]		Retrospective analysis of emergency calls for OHCA	65	57 (where caller responded with an account)	35%	
Schneider et al. 2004 [118]^a^		Survivors of OHCA and people who know them	112	112		*4–5%*
Sipsma, Stubbs & Plorde 2011 [121]		Adults (≥18)	1001	333		*8%*
Swor 2006 [20]		Witnesses of OHCA	684	279 (did not perform CPR)		*4%*
Winkelman et al. 2009 [134]		Teacher candidates	582	47		*2%*
***Lack of confidence***						
Anto-Ocra et al. 2020 [35]		Adults (≥18 years)	277	277		16%
Case et al. 2018 [47]		OHCA Calls	120	120 calls where no CPR given	*“many”*	
Cheskes et al. 2016 [51]		Adult (≥18 yrs)	428	NR	*6–12%*	
Dobbie 2018 [53]		Adults (≥16 years)	1027	1027		15%
Hung 2017 [62]		College and University students (≥15 years)	351	351		48%
Jelinek 2001 [64]		General public (age not reported)	803	26 unwilling to perform CC	*4%*	
Lu et al. 2016 [89]		College students	609	609 (non-medical)		12–40% (dep on subject)
Magid et al. 2019 [92]		College students	588	300 (who identified barriers)		*61%*
		Teachers	383	NR		*49%*
Sasson et al. 2013 [114]		Teachers	383	NR		*49%*
Nishiyama et al. 2019 [100]		University students who had witnessed OHCA	5549	94 (who did not perform CPR)		10%
So et al. 2020 [125]		High school students (12–15 years)	128	NR		91%
Wilks et al. 2015 [133]		Secondary school students (15–16 years)	383	NR		*27%*
***Uncertainty whether cardiac arrest***						
Axelsson et al. 1996 [36]		People who reported making a CPR attempt between 1990 and 1994	742	51 bystanders described hesitation	*NR*	
Breckwoldt et al. 2009 [45]		Witnesses of OHCA	138	39 where agonal breathing	39%	
Case et al. 2018 [47]		OHCA Calls	120	120 calls where no CPR given	*28%*	
Dobbie 2018 [53]		Adults (≥16 years)	1027	1027		14%
Hauff 2003 [59]		Call to Dispatch Centre with OHCA	404	52 who did not accept CPR instructions	*6%*	
Han 2018 [58]		Family members (≥18 years) of patients at risk of cardiac arrest	203	88		10%
Lee et al. 2013 [82]		College students	2029	242 (unwilling to perform CPR)		*34%*
Magid et al. 2019 [92]		College students	588	300 (who identified barriers)		*40%*
Mathiesen et al. 2017 [93]		Witnesses of OHCA	10	10	NR (qualitative barrier identified)	
Mausz, Snobelen & Tavares 2018 [94]		Witnesses of OHCA	14	15	NR (qualitative barrier identified)	
Nishiyama et al. 2019 [100]		University students who had witnessed OHCA	5549	94 (who did not perform CPR)		12%
Nolan et al. 1999 [101]		Adults (≥45)	786	203 (not ready to perform CPR)		*34%*
Platz et al. 2000 [108]		Family members of patients at risk of cardiac arrest	100	100		34%
Sasson et al. 2013 [114]		Lay-people (min age not stated)	42	42	NR (1 of 10 qualitative barriers identified)	
Sasson et al. 2015 [115]		Lay-people (≥13)	64	64	NR (qualitative barrier identified)	
Swor et al. 2013 [126]^a^		Witnesses of OHCA	30	30	10% (seizures/agonal breathing)	
***Feeling unprepared***						
Mausz, Snobelen & Tavares 2018 [94]		Witnesses of OHCA	14	15	NR (qualitative barrier identified)	
Moller et al. 2014 [98]		Witnesses of OHCA	33	33	NR (qualitative barrier identified)	
**13. Emotion**						
***Strong emotions***						
Aaberg et al. 2014 [32]		High School students	399	399 responding as to their worst fear	NR (1 of 3 qualitative themes identified)	
Bohn et al. 2012 [42]		Grammar school pupils (age 10 and age 13)	280	144 (training group)		25%
Case et al. 2018 [47]		OHCA calls	120	120 calls where no CPR given	*20%*	
Dami 2010 [51]		Call to Dispatch Centre with OHCA	738	73 medically appropriate who refused	*42%*	
Hauff 2003 [59]		Call to Dispatch Centre with OHCA	404	52 who did not accept CPR instructions	*11%*	
Iserbyt 2016 [63]		Secondary school pupils	313	313		19% (girls)
						13% (boys)
Kandakai & King 1999 [76]		College students	214	214		61%
Lerner et al. 2008 [83]		Call to Dispatch Centre with OHCA	168	145 who did not follow CPR instructions	*14%*	
Maes et al. 2015 [91]^a^		Hospital visitors (≥13 years)	85	51 who did not feel able to use AED	*4%*	
Mausz, Snobelen & Tavares 2018 [94]		Witnesses of OHCA	14	15	NR (qualitative barrier identified)	
Nishiyama et al. 2019 [100]		University students who had witnessed OHCA	5549	94 (who did not perform CPR)		14%
Platz et al. 2000 [108]		Family members of patients at risk of cardiac arrest	100	100		13%
Riou et al. 2020 [110]		Retrospective analysis of emergency calls for OHCA	65	2	NR (being ‘shaken’ and fear expressed in 2 example quotations)	
Skora & Riegel 2001 [122]		Laypersons who had provided out-of-hospital CPR to strangers	12	12 participants	NR (Qualitative theme identified) Fear and anxiety	
Swor 2006 [20]		Witnesses of OHCA	684	279 (did not perform CPR)		*39%*
Thoren 2010 [113]		Partners of people who experienced OHCA	15	15	NR (Qualitative theme identified)	
Winkelman et al. 2009 [134]		Teacher candidates	582	47		*13%*
***Embarrassed***						
Lu et al. 2016 [89]		College students	609	609 (non-medical)		4–32% (dep on subject)
**12. Social influences**						
***Reluctance to take responsibility / get involved***						
Lu et al. 2016 [89]		College students	609	609 (non-medical)		3–64% (dep on subject)
Nishiyama et al. 2019 [100]		University students who had witnessed OHCA	5549	94 (who did not perform CPR)		6%
Sasson et al. 2013 [114]		Lay-people (min age not stated)	42	42	NR (1 of 10 qualitative barriers identified)	
***Wait for someone else to step forward***						
Johnston 2003 [65]		Adults (≥18 years)	4490	4490	2%	
Magid et al. 2019 [92]		College students	588	300 (who identified barriers)		*20%*
***Believe should wait for health professional***						
Huang 2016 [60]		School and University students (13–21 years)	1407	546 (unwilling to perform on stranger)		*7%*
Kua et al. 2018 [79]		School students (11–17 years)	1196	966		28%
Pei-Chuan Huang et al. 2019 [107]		Adults (≥20)	1073	141 (who provided reasons why not)		*3.5%*
Tang et al. 2020 [127]		High school students (senior, age NR)	397	397		33%
***Perceptions about what others would do?***						
Sasson et al. 2013 [114]		Lay-people (min age not stated)	42	42	NR (1 of 10 qualitative barriers identified)	
***Modesty concerns***						
Becker et al. 2019 [39]		Adults (≥18 years) who attended CPR training event	677	249 resp. concerns for woman	14%	
Shams et al. 2016 [119]		College students	948	948		18% chest exposure
						10% touching opposite gender
***Reluctance to touch a stranger***						
Babic et al. 2020 [38]		Adults (≥18 years)	198	198		10% (MMV)
						5% (compressions)
Becker et al. 2019 [39]		Adults (≥18 years) who attended CPR training event	677	306 resp. concerns elderly patient	2% (or blame)	
				249 resp. concerns for woman	6% (or be accused)	
				291 resp. concerns for child	5% (blame)	
**11. Environmental context**						
***Disagreeable characteristics***						
***General***						
Axelsson et al. 1996 [36]	People who reported making a CPR attempt between 1990 and 1994	742	51 bystanders described hesitation	*NR*		
Dobbie 2018 [53]	Adults (≥16 years)	1027	1027		19%	
Hauff 2003 [59]	Call to Dispatch Centre with OHCA	404	52 who did not accept CPR instructions	*2%*		
Lerner et al. 2008 [83]	Call to Dispatch Centre with OHCA	168	145 who did not follow CPR instructions	*3%*		
Shams et al. 2016 [119]	University students	948	948		30%	
***Blood***						
Johnston 2003 [65]	Adults (≥18 years)	4490	4490	12%		
Lester, Donnelly & Weston 1997 [84]	First year high school pupils	233	233	23%		
Lester, Donnelly & Assar 1997 [85]	General public	241	241	5%		
Cu 2009 [50]	Caregivers of children presenting to the Emergency Department (≥18 years)	348	125 (unwilling to perform CPR on adult)		*10%*	
Kandakai & King 1999 [76]	College students	214	214		88%	
Skora & Riegel 2001 [122]	Previously performed CPR	12	12 participants	NR (Qualitative theme identified)		
***Dirty***						
Dobbie 2018 [53]	Adults (≥16 years)	1027	1027		5%	
Johnston 2003 [65]	Adults (≥18 years)	4490	4490	11%		
***Vomit***						
Johnston 2003 [65]	Adults (≥18 years)	4490	4490	3%		
Kandakai & King 1999 [76]	College students	214	214		81%	
Nolan et al. 1999 [101]	Adults (≥45)	786	203 (not ready to perform CPR)		*38%*	
Skora & Riegel 2001 [122]	Previously performed CPR	12	12 participants	NR (Qualitative theme identified) momentary hesitation		
***Saliva***						
Kandakai & King 1999 [76]	College students	214	214		54%

**Table 4:** Psychological and behavioural factors associated with GREATER actual/intended CPR initiation (grouped by Theoretical Domains Framework V.2 [29])

Factors related to initiation of CPR	Participants	Number (total)	Number in analysis for this factor	Unprompted identification of each factor (% of whole sample and % of *unwilling subsample*)	Endorsement of each factor when prompted (% of whole sample and % of *unwilling subsample*)
**3. Social role and identity**					
***Instinct for saving others***					
Huang 2016 [70]	School and University students (13–21 years)	1407	807 (willing to perform on stranger)		*89%*
***Sense of personal responsibility/duty***					
Huang 2016 [70]	School and University students (13–21 years)	1407	807 (willing to perform on stranger)		*64%*
Kua et al. 2018 [79]	School students (11–17 years)	1196	966		34%
Mathiesen 2017 [93]	Witness of OHCA	10	10	NR (Qualitative theme identified - normative obligation)	
Skora 2001 [122]	Previously performed CPR	12	12	NR (Qualitative themes identified – Duty & Responsibility, Guilt and Social pressure, Altruism)	
Wilks 2015 [133]	Secondary school students (15–16 years)	383	NR		*NR*
**6. Beliefs about Consequences**					
***Anticipate guilt if don’t act***					
Mathiesen 2017 [93]	Witness of OHCA	10	10	NR (Qualitative theme identified)	
***Believe more likely to help than harm***					
Hung 2017 [72]	College and University students (≥15 years)	351	351		79%
Kua et al. 2018 [79]	School students (11–17 years)	1196	966		12%
Pei-Chuan Huang 2019 [107]	Adults (≥20 years)	1073	1073		85%
***Person will die if I don’t***					
Johnston 2003 [75]	Adults (≥18 years)	4490	4490	6%	
***Believe CPR increases survival***					
Hung 2017 [72]	College and University students (≥15 years)	351	351		79%
Wilks 2015 [133]	Secondary school students (15–16 years)	383	NR		*NR*
***Know risk of permanent brain damage if don’t act***					
Pei-Chuan Huang 2019 [107]	Adults (≥20 years)	1073	1073		79%
Johnston 2003 [75]	Adults (≥18 years)	4490	4490	6%	
Kua 2018 [79]	School students (11–17 years)	1196	966	37%	
***Awareness of legal protection (*e.g.*Good Samaritan Law)***					
Pei-Chuan Huang 2019 [107]	Adults (≥20 years)	1073	1073		85%
**12. Social influences**					
***Make every effort even if no hope***					
Huang 2016 [70]	School and University students (13–21 years)	1407	807 (willing to perform on stranger)		*13%*
***Belief that life is precious***					
Hung 2017 [72]	College and University students (≥15 years)	351	351		49%
Mathiesen 2017 [93]	Witness of OHCA	10	10	NR (Qualitative theme identified)

**Table 5:** Studies which formally assess association of variables with measures of CPR initiation/intention (grouped by Theoretical Domains Framework V.2 [29])

**Factor associated with CPR initiation**	**Population (Number, Country, Age Group)**	**Measure of CPR intention**		**Variable associated with CPR initiation**	**Odds ratio (95% CI) (unless indicated otherwise)**
**1. Knowledge**					
***Knowing importance of CPR***					
Kuramoto 2008 [80]	1132 Japan Adults (≥15 years)	Willingness to attempt CPR			1.9 (1.3–2.8)
**11. Environmental context**					
***Having friends with heart diseases***					
Kuramoto 2008 [80]	1132 Japan Adults (≥15 years)	Willingness to attempt CPR			1.8 (1.1–3.0)
***Self-rated health status***					
Ro et al. 2016 [111]	62,425 Korea ≥19 years	Provision of bystander CPR (CPR self-efficacy)		Good self-rated health status	1.3 (1.2–1.4)
**2. Skills**					
***Previous experience of CPR***					
Chew et al. 2019 [53]	6248 Malaysian Adults (min age NR)	Willingness to perform CPR		Previous experience of administering CPR	Mean rank =2877.42, U = 1,205,596, *p* < 0.001
Hawkes et al. 2019 [68]	2084 UK Adults (≥18 years)	Likelihood of performing CPR		Having witnessed OHCA previously	1.53 (1.17–2.10)
Kuramoto 2008 [80]	1132 Japan Adults (≥15 years)	Willingness to attempt CPR		Actual experience with CPR	3.8 (1.7–8.)
Sasaki et al. 2015 [113]	4853 Japan adults (≥15 years)	Confidence in performing CPR		Previous experience performing CPR	CC: 4.8 (1.8–12.9)
					MMV: 3.7 (2.1–6.6)
					AED: 2.7 (1.3–5.7)
Schmid et al. 2016 [116]	371 Costa Rica age unknown	Willingness to perform CPR on a stranger		Prior witness OHCA	2.5 (1.2–5.3)
**6. Beliefs about Consequences**					
***Believe legal consequences if person dies***					
Schmid et al. 2016 [116]	371 Costa Rica age unknown	Willingness to perform CPR on a stranger		Belief that CPR has legal consequences	0.4 (0.2–0.6)
***Hesitancy about mouth to mouth***					
Schmid et al. 2016 [116]	371 Costa Rica age unknown	Willingness to perform CPR on a stranger		Hesitancy to do MMV	0.3 (0.2–0.6)
***Outcome expectancies***					
Meischke et al. 2002 [97]	159 USA older adults	Intentions to use an AED	Outcome expectancies		4.65 (2.0–10.6)
***Attitudes***					
Vaillancourt et al. 2013 [131]	192 Canada Adults (≥55 years)	Intention to perform CPR	Attitude		1.6 (1.3–2.0)
Magid et al. 2019 [92]	588 USA College students	Intention to perform CPR	Attitude		Beta (95%CI): 0.164 [0.131, 0.197]
**4. Beliefs about capabilities**					
***Feeling confident in ability to perform CPR***					
Shams et al. 2016 [119]	948, Lebanon, university students	Willingness to perform CPR		Feeling confident in abilities	1.9 (1.3–2.9)
				Feel lack expertise	0.6 (0.4–0.8)
Meischke et al. 2002 [97]	159 USA older adults	Intentions to use an AED		Self-perceived ability	11.5 (3.8–34.4)
Vaillancourt et al. 2013 [131]	192 Canada Adults (≥55 years)	Intention to perform CPR		Control	1.4 (1.2–1.5)
Magid et al. 2019 [92]	588 USA College students	Intention to perform CPR		Perceived Behavioural Control	Beta (95%CI): 0.083 [0.047, 0.119]
**12. Social influences**					
Vaillancourt et al. 2013 [131]	192 Canada Adults (≥55 years)	Intention to perform CPR		Normative	1.2 (1.1–1.4)
Magid et al. 2019 [92]	588 USA College students	Intention to perform CPR		Subjective norm	Beta (95%CI): 0.176 [0.133, 0.219]
**Studies Reporting differences in beliefs between participants who were willing to perform CPR and those who were unwilling**					
		**Group**		**Belief (measure)**	**Difference between the groups**
		**willing**	**unwilling**		
**4. Beliefs about Capabilities**					
Nolan et al. 1999 [101]	786 Canada Adults (≥45)	62%	47%	Self-efficacy (confidence to perform CPR)	*P* < 0.001
Schmitz et al. 2015 [117]	110 (experimental group)	11.1	8.6	Self-efficacy (capacity belief) (self-efficacy score (higher score = greater efficacy)	*P* = 0.009
**6. Beliefs about Consequences**					
Parnell 2006 [106]	494 New Zealand High School Students	70% positive attitude	47% negative attitude	Attitudes (% positive or negative attitude)	*P* < 0.001
Schmitz et al. 2015 [117]	110 (experimental group)	22.3	18.8	Attitudes (attitude score (higher score = more positive attitude)	*P* = 0.04
**13. Emotion**					
Nolan et al. 1999 [101]	786 Canada Adults (≥45)	2.17	2.42	Anticipate negative emotions with CPR (mean number of negative emotions))	*P* < 0.02

**Table 6:** Summary of studies exploring relationship to victim (Domain 11. Environmental context and resources [29])

Author	Country	Participants	n	Relatives (%)	Neighbour/Friend (%)	Unknown person (%)	Drug addict (%)	Unkempt (%)	Difference (%)	Other statistics
Alhussein 2021 [33]	Saudi Arabia	Adults (≥18)	413 (subsample aware of CPR)	36	24 /22	16			20	*P* < .001
Anto-Ocra et al. 2020 [35]	Ghana	Adults (≥18) not medical	277	78	60	46			32	
Axelsson 2000 [37]	Sweden	Adults (≥18 years) who had received training in basic CPR in January 1997.	1012	97	91	70	17	7	27	
Bin 2013 [40]	Saudi Arabia	High school students	575	67 (male respondents)		42 (male respondents)			25	
				67 (female respondents)		24 (female respondents)			43	
Birkun 2018 [41]	Crimea	Adult (≥18 yrs)	384	91		79			12	
Bray 2017 [44]	Australia	Adult (≥18 yrs)	404	91 (conventional CPR, low rate area)	88 (conventional CPR, low rate area)				3	
Brinkrolf 2018 [46]	Germany	Witnesses of an OHCA	101	70.20	60	59.40			11	
Chen 2017 [48]	China	Adult laypersons (≥18 yrs) + 3 < 18 years	1841	98.7		76.3			22.4	
Cheng 1997 [49]	Taiwan	Families of cardiac patients and general public	856	92.40	88	75.10			17.3	
Cheng-Yu 2016 [50]	Taiwan	Adults (≥20 years)	401		86.80	36.60			50	
Chew 2009 [52]	Malaysia	School teachers.	73	97.30	94.50			8.20		
Cho 2010 [54]	Korea	Lay people aged 11 years and over	890	55.80		19			36	
Coons 2009 [56]	USA	Adult (≥18 years)	370 (urban)	84.5 (urban)		51.3 (urban)			33	
			385 (rural)	82.5 (rural)		55 (rural)			28	
Cu 2009 [57]	Australia	Caregivers of children presenting to the Emergency Department (≥18 years)	348	81		64			17	*P* < 0.001
De Smedt 2018 [59]	Belgium	Schoolchildren aged 10–18, teachers and principals	390	96	92	67 (woman)			29	
Dracup 1994 [62]	USA	Family members of patients at risk of cardiac arrest	172	86		82			4	
Fratta et al. 2020 [65]	USA	Attendees at large public gatherings (aged ≥14)	516	69		45				*P* < 0.001
Han 2018 [66]	Korea	Family members (≥18 years) of patients at risk of cardiac arrest	203	68 (CS group)		64 (CS)			4	
				76 (CV group)		65 (CV)			5	
				67 (no risk group)		50 (no risk)			17	
Hollenberg et al. 2019 [69]	Sweden	School students (13 years)	641		85 (directly after training native)			38 (directly after training native)	47	NR
					84 (Directly after training other native)			52 (Directly after training other native)	32	
					78 (at 6 mths native)			31 (at 6 mths native)	47	
					80 (at 6 months other native			42 (at 6 months other native	38	
Iserbyt 2016 [73]	Belgium	Secondary school pupils	313	51 (F)	49 (F)	11 (F)			40 (F)	All scores increased with training
				49 (M)	48 (M)	8 (M)			41 (M)	
Jelinek 2001 [74]	Australia	General public (age not recorded)	803		96 (trained < 12 months)	54.5 (trained < 12 months)			42	
					94.4 (trained 1–5 y)	51.8 (trained 1-5y)			43	
					90 (trained ≥5y)	45.2 (trained≥5 y)			45	
Karuthan et al. 2019 [78]	Malaysia	College students	393	68		55			13	
Kuramoto 2008 [80]	Japan	Adults (≥15 years)	1132	13		7			6	
Lam 2007 [81]	Hong Kong	Laypersons who attended the CPR course (aged ≥7 years)	305	87		61			26	
Lester 1997b [85]	Wales	General public	241	Adult 100 (definitely or probably)	100	99			1	
Locke 1995 [88]	USA	Lay people (minimum age not reported) & health care providers	975	94		55			39	
Mecrow 2015 [96]	Bangladesh	Lay people (≥10 years)	721	Data extracted for mother	Data extracted for friend of same sex					
				88 (M)	80.8 (M)	50 (M)			38 (M)	
				96.4 (F)	75.3 (F)	47 (F)			49 (F)	
Nord 2016 [102]	Sweden	Schoolchildren	1124		75 (App training grp)	32 (App training grp)			43 (App)	
					78 (DVD training gps)	31 (DVD training gp)			47 (DVD)	
Nord 2017 [103]	Sweden	Schoolchildren	549		76 (O training grp)	31 (O training grp)			45 (O)	
					73 (T training grp)	31 (T training grp)			42 (T)	
					78 (RT grp)	29 (RT training grp)			49 (RT)	
Omi 2008 [104]	Japan	High school students	3316	41		15			26	
Parnell 2006 [106]	New Zealand	High school students	494	84		63			21	
Pei-Chuan Huang 2019 [107]	Taiwan	Adults (≥20 years)	1073	92		86.7 (assuming skill)				
Rankin et al. 2020 [109]	Australia	Adults (18–21 years)	178	82		64			18	
Rowe 1998 [112]	Canada	Adults (≥44 years)	811	58		41			17	
Shibata 2000 [120]	Japan	High school students and teachers	479	52.8 (students) CC + MMV		12.9 (students) CC + MMV			40 (students MMV)	
			147	63.9 (teachers) CC + MMV		25.2 (teachers) CC + MMV			29 (teachers MMV)	
			479	84.8 (students) CC only		73.1 (students) CC only			12 (students CC-only)	
			147	89.8 (teachers) CC only		75.5 (teachers) CC only			14 (teachers CC-only)	
So et al. 2020 [125]	Hong Kong	High school students (12–15 years)	128	25	24	18			7	
Taniguchi 2007 [128]	Japan	High school students and teachers	3444	41.1 (students)		14.8 (students)			26 (students)	
				64.5 (teachers)		28.5 (teachers)			36 (teachers)	
Taniguchi 2012 [129]	Japan	High school students and teachers	1946	42 (students MMV)		16 (students MMV)			26 (students)

**Table 7:** Studies exploring relationship with victim (Likert Scale) ((Domain 11. Environmental context and resources [29])

					Median (IQR)		Sig. level
Study	Country	Participants	Number of participants	Willingness measured on likert scale	Friend/family	Stranger	
Bouland 2017 [43]	USA	Laypeople (≥14 years)	238	1–10	9 (5–10)	5 (3–8)	*p* < 0.001
					**Mean (SD)**		
Lynch 2010 [90]	USA	Adults (40–70 years)	822	1–5	4.06 (1.18)	3.68 (1.23)	NR
Sneath 2009 [124]	USA	Adults (≥18 years)	78	1–5	4.01 (NR)	2.74 (NR)	NR

**Table 8:** Studies exploring mouth-to-mouth ventilation as a deterrent (Domain 6. Beliefs about consequences [29])

Study	Country	Participants	Sample (n)	CPR inc. ventilations	At least CC-only	% of sample more likely to do CC-only CPR	Significance (if stated)
Bray 2017 [44]	Australia	Adult (≥18 yrs)	404	91% (close family)	91% (close family)	0 (family)	
			(results from 223 in low-bystander region)	88% (friend)	91% (friend)	3 (friend)	
				67% (stranger)	88% (stranger)	21 (stranger)	
Cheng-Yu 2016 [50]	Taiwan	Adults (≥20 years)	401	86.8% (known)	88.1% (known)	1.3 (known)	
				36.6% (stranger)	67.8% (stranger)	31.2 (stranger)	
Cheskes 2016 [51]	Canada	Adult (≥18 yrs)	428	39.7% (stranger)	61.5% (stranger)	21.8 (stranger)	(61.5% v. 39.7%, *p* < 0.001).
Cho 2010 [54]	Korea	Lay people aged 11 years and over	890	55.8% (family)	55.5% (family)	0.3 (family)	
				19% (adult)	30.1% (adult)	11.1 (adult)	
Enami 2010 [64]	Japan	Adults (≥17 years). New driver licence applicants	8890	72%	86.3%	14.3	
Hubble 2003 [71]	USA	High school students	683	43%	55%	12	*P* < 0.001
Jelinek 2001 [74]	Australia	General public (age not recorded)	803	90.7% (friend/relative)	91.4% (friend/relative)	0.7 (friend/relative)	
				47.2% (stranger)	78.1% (stranger)	30.9 (stranger)	
Lam 2007 [81]	Hong Kong	Laypersons who attended the CPR course (aged ≥7 years)	305	87% (family)	93% (family)	6 (family)	
				61% (stranger)	84% (strangers)	23 (stranger)	
Lester 2000 [86]	UK	Participants who had attended a CPR course	416	82%	94%	12	
Locke 1995 [88]	USA	Lay people (minimum age not reported) & health care providers	975	74% (friend/relative)	88% (friend/relative)	14 (friend/relative)	
				15% (stranger)	68% (stranger)	53 (stranger)	
Nielsen 2013 [99]	Denmark	Adults (≥15 years)	1639	59% (stranger)	63% (stranger)	4	
Nord 2016 [102]	Sweden	Schoolchildren	1124	75% (known-App grp)	93% (known-App)	18 (known)	
				78% (known DVD grp)	94% (DVD grp)	16 (known)	
				32% (stranger –app)	87% (stranger-app)	55 (stranger)	
				31% (stranger DVD)	82% (stranger DVD)	51 (stranger)	
Nord 2017 [103]	Sweden	Schoolchildren	397	73% (friend-T grp)	92% (friend T grp)	19 (friend T grp)	
				78% (friend RT grp)	98% (friend RT grp)	20 (friend RT grp)	
				31% (stranger- T grp)	83% (stranger –T grp)	52 (stranger)	
				29% (stranger- RT grp)	87% (stranger RT grp)	58 (stranger)	
Omi 2008 [104]	Japan	High school students	3316	41% (relative)	69% (relative)	28 (relative)	
				15% (stranger)	53% (stranger)	38 (stranger)	
Smith 2003 [123]	Australia	Householders (age not reported)	1489	60.5% (stranger)	79.7% (stranger)	19.2 (stranger)	
Shibata 2000 [120]	Japan	High school students and teachers	626	Students (*n* = 479)	Students	Students	Students
				12.9% (strangers)	73.1% (strangers)	60.2 (stranger)	*p* < 0.001
				52.8% (relatives)	84.8% (relatives)	32.0 (relatives)	*p* < 0.001
				Teachers (*n* = 147)	Teachers	Teachers	Teachers
				25.2% (strangers)	75.5% (strangers)	50.3 (stranger)	*p* < 0.001
				63.9% (relatives)	89.8% (relatives)	25.9 (relatives)	*p* < 0.001
Taniguchi 2012 [129]	Japan	High school students and teachers	1946	Students (*n*=1708)	Students	Students	
				42% (relative)	72% (relative)	30 (relative)	
				16% (stranger)	59% (stranger)	43 (stranger)	
				Teachers (*n* = 238)	Teachers	Teachers	
				60% (relative)	84% (relative)	24 (relative)	
				28% (stranger)	73% (stranger)	45 (stranger)

**Table 9:** Studies exploring mouth-to-mouth ventilation as a deterrent (Likert Scale) ((Domain 6. Beliefs about consequences [29])

Study	Country	Participants	N^**o**^ of participants	Willingness (likert scale)	Type of CPR (median)		Sig. level
					CC & ventilation	CC only	
Vetter 2016 [132]	USA	High school students	412	1–5 (1 most likely)	2 (relative)	1.6 (relative)	*P* < 0.02

**Table 10:** Studies exploring disagreeable characteristics (Domain 11. Environmental context and resources [29])

Study	Country	Participants	Sample (n)	% willing to perform CPR in presence of disagreeable characteristic	Adult stranger	Difference
				***Vomit***		
Lester, Donnelly & Assar 1997 [85]	Wales	General public	241	25%	69%	44%
Smith et al. 2003 [123]	Australia	Householders (age not reported)	1489	73% (CC)	80% (CC)	7% (CC)
				41.5% (MMV)	60.5% (MMV)	19% (MMV)
				***Not Clean***		
Lester, Donnelly & Assar 1997 [85]	Wales	General public	241	30%	69%	39%
Smith et al. 2003 [123]	Australia	Householders (age not reported)	1489	75% (CC)	80% (CC)	5% (CC)
				52% (MMV)	60.5 (MMV)	8.5% (MMV)
				***Smells***		
Lester, Donnelly & Assar 1997 [85]	Wales	General public	241	30%	69%	39%
				***Bleeding***		
Lester, Donnelly & Assar 2000 [86]	UK	Participants who had attended a CPR course	*n* = 365 (facial blood)	68% (CC)	94% (CC)	26% (CC)
			*n* = 367 (adult stranger)	40% (MMV)	82% (MMV)	42% (MMV)
Smith et al. 2003 [123]	Australia	Householders (age not reported)	1489	55% (CC)	80% (CC)	25% (CC)
				39% (MMV)	60.5% (MMV)	21.5% (MMV)

### Studies involving those with direct experience of OHCA

Sixteen studies involving people with direct experience of OHCA were identified. These included five studies which analysed recorded calls involving OHCA [47, 58, 67, 83, 110], four qualitative studies exploring the experiences of people who had witnessed an OHCA [93, 94, 98, 130] and seven cross-sectional surveys which asked open questions about people’s experiences of facilitators and barriers to them having performed CPR [20, 36, 46, 95, 122, 126, 136].

#### Real-life calls

##### TDF domain 4: beliefs about capabilities

Limitations in the *physical capacity* of the caller was also identified in all five studies. Physical capability was a barrier to CPR in 15% [47], 51% [58], 11% [67], 35% [110] and 8% [83] of calls. Difficulties moving the person who had collapsed to a flat position in order to perform CPR and the rescuer being frail or with a condition making CPR difficult were described. *Uncertainty about whether cardiac arrest was happening* (e.g. person still making some respiratory sounds) was reported in 28% of calls by Case (2018) [47] and in 6% by Hauff (2003) [67].

Case (2018) [47] reported that “many callers” reported a *lack of confidence*.

##### TDF domain 6: beliefs about consequences

Concerns that *CPR was futile* (e.g. that the person was already dead/beyond help) were reported in 50% of calls analysed by Riou et al. (2020) [110], in 28% of calls analysed by Case (2018) [47] and in 23% by Hauff (2003) [67]. *Concern about infection* (4%) [58], *fear of doing harm* (3%) and *fear of legal consequences* (1%) [83] were reported in a small minority of calls.

##### TDF domain 11: environmental context

*Disagreeable characteristics* associated with the victim was identified as a factor in 3% [83] and 2% [67] of calls.

##### TDF domain 13: emotion

All five studies of real-life calls analysed calls where the layperson hesitated or refused to provide CPR identified the *strong emotion of the situation* as a factor that prevented initiation of CPR. Elements of emotional distress, such as panic, upset and stress were identified in 20% [47], 42% [58], 11% [67] and 14% [83] of calls where callers expressed reluctance. ‘Being shaken’ and ‘fear’ were described in 2 example quotations by Riou et al. (2020) [110].

#### Qualitative studies of people who have witnessed OHCA

Four qualitative accounts of people’s experiences of encountering OHCA and CPR were identified [93, 94, 98, 130] comprising interviews with a total of 107 participants (aged 24 [93] to 87 [130]).

##### TDF domain 2: skills

*Feeling unprepared* as to what to expect in a cardiac arrest was a theme identified by Mausz (2018) [94] and Moller (2014) [98], in particular that reality was very different from training with a manikin [98].

##### TDF domain 3: social/professional role and identity

A *sense of community or social responsibility* were described as *encouraging* performance of CPR, some stating it was expected of any responsible citizen [93].

##### TDF domain 4: beliefs about capabilities

Problems *identifying whether cardiac arrest had actually occurred* (and thus whether CPR was indicated) were identified [93, 94].

##### TDF domain 6: beliefs about consequences

*Fear of doing the patient harm* was identified as a cause for hesitation [130]. Recognising the extreme seriousness of the situation led people to erroneously assume that the person was already dead and that *CPR would be futile* [130]. However, anticipating feeling guilty if they didn’t perform CPR and the person died as a result was a motivation for participants [93].

Concerns about personal safety [93] and liability in the context of a workplace [94] were also expressed.

##### TDF domain 11: emotion

Participants also described experiencing *panic and extreme emotions* which inhibited their ability to perform CPR actions [94, 130].

#### Cross-sectional surveys

Eight cross sectional surveys included analyses of barriers and facilitators of CPR identified by participants who had direct experience of OHCA [20, 46, 93, 95, 100, 122, 126, 136]. Issues identified were very similar to those already described above in the qualitative studies:

### Studies of participants where direct experience of CPR was not required

#### Studies examining the relationship between psychological/behavioural variables and willingness/confidence/intention to perform CPR

Thirteen studies formally explored the relationship between behavioural and psychological predictor variables and willingness to initiate CPR (see Table 5).

##### TDF domain 1: knowledge

Knowing the importance of CPR (OR 1.9) was positively and significantly related to willingness to perform CPR [80].

##### TDF domain 2: skills

Having *previous experience of CPR or OHCA* was the strongest predictor of anticipated willingness to perform CPR [80, 113, 116]. Odds ratios across four studies ranged from 1.5 [68] to 4.8 [113].

##### TDF domain 4: beliefs about capabilities

Those with *good self-rated health status* (AOR, 1.26) were more likely to report that they could provide bystander CPR than those reporting poor health [111] and *feeling confident* (OR 1.9) [119] was positively and significantly related to willingness to perform CPR [97]. *Perceiving a lack of expertise* was negatively related to willingness (OR 0.6) [119]. Nolan et al. (1999) [101] also showed that *confidence* differed significantly between those willing and unwilling to initiate CPR. Those unwilling to act also perceived a greater number of psychosocial barriers than those willing (*p* ≤ .05).

Vaillancourt (2013) [131] and Magid (2019) [92] explored the ability of constructs from the Theory of Planned Behaviour, to predict intention to perform CPR in the event of a cardiac arrest. *Attitudes* (e.g. I could save someone’s life with CPR) were with the strongest predictor of respondents’ intentions to perform CPR (OR 1.63) identified by Vaillancourt (2013) and also found to be significant in predicting intention to perform CPR. Similarly, both Vaillancourt (2013) and Magid (2019) found higher *control beliefs* (I feel confident in my abilities to perform CPR) to be significantly related to increased intentions to perform CPR on a cardiac arrest victim (OR 1.16) [131].

##### Domain 12: social influences

Normative beliefs (derived from the Theory of Planned Behaviour (TPB)) (e.g. My friends and family expect me to do CPR) were found to be modestly but significantly related to people’s intentions to perform CPR on a cardiac arrest victim (OR: 1.07) [131]. Magid (2019) [92] also found subjective norms predictive of intention to perform CPR.

##### TDF domain 13: emotion

Nolan et al. (1990) [101] showed that confidence differed significantly between those willing and unwilling to initiate CPR. Those unwilling to act anticipated a higher number of negative emotions (afraid, sad, angry, anxious, confused) if they were to perform CPR compared to those who were willing to act (*p* ≤ .02).

#### Studies which have compared responses to scenarios -varying psychological/behavioural factors

Sixty-two studies explored a variety of other factors related to willingness to perform CPR (see Tables 6, 7, 8, 9 and 10). Respondents were more willing to perform CPR on their family and friends compared to strangers and in situations that did not involve disagreeable characteristics (TDF Domain 11: Environmental context and resources).

Respondents were more willing to perform compression-only CPR compared to mouth-to-mouth CPR and in situations where there was a perceived risk of transmissible infection willingness to perform CPR was reduced, e.g. after a SARS outbreak (TDF Domain 6: Beliefs about consequences).

#### Studies of people’s anticipated barriers and facilitators to CPR

##### Qualitative studies

Four studies provided qualitative accounts of people’s perceptions of CPR [61, 114, 115, 135]. Many of the barriers anticipated by participants in these studies were similar to those identified by people with direct experience of OHCA, as reported above. Additionally, issues around a general fear of ‘getting involved’ with possible consequences in relation to immigration status/law enforcement [115] were identified (TDF Domain 6: Beliefs about Consequences).

##### Cross-sectional data

Twelve studies [32, 39, 51, 60, 71, 73–75, 84, 85, 91, 104] explored the reasons people indicated a reluctance or unwillingness to perform CPR using open questions (rather than presenting possible reasons).

- Unprompted reasons provided by those categorised as ‘unwilling’
- *TDF domain 4: beliefs about capability* Concerns around capability were reported by 11% of unwilling high school students [104], and by 45% of those not willing to use an AED [91]. Concerns about physical capability in particular were reported by 11% of unwilling general public [74]. Low confidence was also reported (4%) [74] and 6–12% [51].
  *TDF domain 6: beliefs about consequences* The reasons most commonly volunteered by those categorised as unwilling were concerns about to the risk to self: 56% of unwilling general public [74] with 24% [51], 35% [71] and 19% [74] concerned about the risk of infection in particular. Concerns about doing harm to the casualty were reported by 25% [71] and 23% [104]. Legal concerns were reported by 13% [71] and 19% [74] of people unwilling to provide CC and by 16% [71] and 4% [74] of those unwilling to provide mouth-to-mouth ventilation. CPR violating beliefs about death were also reported (4%) [71].
  *TDF domain 13: emotion* Being too stressed (4%) [91] was also reported as a reason for unwillingness.

- Prompted reasons
- The reasons for not performing CPR most commonly proposed by researchers were: fear of doing harm (27 studies); concerns about infection (29 studies); legal concerns (24 studies); concerns about capability (26 studies) and concerns about mouth-to-mouth ventilation (10 studies). Averaging across the studies, the reasons endorsed by the largest proportion of unwilling participants were *Lack of confidence* (TDF Domain 4: Beliefs about capabilities), *Fear of doing it wrong* (TDF Domain 6: Beliefs about consequences) and *Concerns about capability* (TDF Domain 4: Beliefs about capabilities)*.*

## Discussion

We have conducted a comprehensive, high-quality, pre-registered systematic review of the psychological and behavioural factors relating to initiation of CPR. This provides a useful synthesis of the evidence to date and identifies promising avenues for intervention and further research. The prominence of two themes: the *overwhelming emotion of the OHCA situation* and *concerns about physical capability* in the more methodologically strong studies [58, 83] and evident across the various designs suggests these may be particularly important to address in order to increase CPR initiation.

### Emotion of the situation

All five studies [47, 58, 67, 83] that analysed call-recordings involving actual CPR attempts identified the emotion of the situation as an important factor delaying initiation of CPR, as did studies of people who had witnessed OHCA [20, 94, 130].

In hypothetical studies, the expectation of high emotion was significantly associated with not being prepared to act [101] and identified as a likely barrier to CPR by high school students [32]. However, interestingly the potential impact of strong emotions was not frequently anticipated by those without experience of CPR (even when prompted) suggesting people may under-estimate the impact of emotion on their behaviour. Helping people to prepare for the unanticipated impact of strong emotions and providing strategies to perform CPR despite their emotional response might be helpful.

### Concerns about capability

Concerns about physical capability were identified as a barrier to initiation in all five studies that analysed emergency call recordings [47, 58, 67, 83], identified in a survey of people who had witnesses an OHCA [20] and provided unprompted as an issue by 11% of the general public [74]. Further, those with good self-rated health were more likely to report being able to perform CPR than those with poor health [111]. Evidence also identified that feeling confident about one’s capability [119] and self-perceived capability [97, 117] are associated with increased willingness to perform CPR and conversely that a lack of confidence reduces willingness [101]. Concerns about capability were identified unprompted by 11% of students [104] and endorsed when prompted by up to 80% of participants. This triangulation of evidence from very different sources suggests concerns about capability as a key issue. Concerns may reflect actual physical limitations amongst potential rescuers but are also likely to reflect people’s beliefs about their capabilities; both are amenable to intervention but importantly will require very different approaches.

### Predictors of CPR that have been formally tested

Studies which statistically tested the relationship between variables of interest and intention to perform CPR or actual behaviour were few, highlighting a need for more definitive studies to confirm posited relationships. Previous experience in performing [80, 113] or witnessing CPR [116] and self-perceived ability [97] were the variables most strongly associated with willingness suggesting interventions that improve perceptions of capability may be helpful.

Six studies found evidence to support predictors derived from behavioural theory such as the Theory of Planned Behaviour [137], highlighting the potential utility of an approach to intervention that is based on behavioural theory. Positive attitudes about CPR [92, 106, 131], perceived behavioural control [92, 131] and normative beliefs [92, 131] were significantly associated with intention to perform CPR and Magid (2019) [92] found the theory accounted for 51% of the variance in intention to perform CPR overall. These belief-based constructs are amenable to change and thus are promising targets for intervention. Resources such as the Behaviour Change Technique Taxonomy [138] and the Theory and Techniques resource (https://theoryandtechniquetool.humanbehaviourchange.org/) are available to help researchers and practitioners identify techniques to include in interventions based on their likely mode of action and their likely effectiveness to change the behaviour of interest (in this case initiation of CPR) in the required situation of OHCA.

Overall, it was notable how few papers explicitly discussed underlying theory and how multiple terms were used to refer to highly similar constructs (e.g. intention, willingness, readiness, prepared to act, capable in an emergency). Construct proliferation [139] and lack of precision in defining and labelling of constructs limits our collective ability to synthesise available evidence and to build a cumulative science [140]. This may lead to wasteful duplication of effort and hinder our ability to identify factors that increase initiation of CPR and, importantly, the factors that make initiation of CPR less likely. Greater attention to robust study design, explicit use of theory or at least consistent definitions of terms might bring us more quickly to our collective goal of increasing CPR initiation.

### Limitations

This review is limited as we have only assessed published materials. There is thus the potential that publication bias has resulted in studies with negative findings being less likely to be identified [141]. We identified a preponderance of cross-sectional surveys using unvalidated measures with relatively little formal testing of posited ‘predictors’ meaning that it is difficult to draw robust and reliable conclusions from the literature.

## Conclusion

Many psychological and behavioural factors associated with CPR initiation can be identified from the current literature with varying degrees of supporting evidence. Preparing people to manage strong emotions and increasing their perceptions of capability are likely important foci for interventions aiming to increase CPR initiation.

Greater use of theory and more robust study designs would strengthen knowledge in this area.

PROSPERO registration number: CRD42018117438.

## Supplementary Information

**Additional file 1. **Search strategy.

## Data Availability

The datasets used and/or analysed during the current study are available from the corresponding author on reasonable request.

## Abbreviations

CPR: Cardio-pulmonary resuscitation
OHCA: Out of hospital cardiac arrest
EPHPP: Effective public health practice project
QARI: Qualitative assessment and review instrument
PRISMA: Preferred reporting items for systematic review and meta-analysis
TPB: Theory of planned behaviour
TDF: Theoretical domains framework
