# Supplementary material for: The psychological and behavioural factors associated with laypeople initiating CPR for out-of-hospital cardiac arrest: a systematic review
Source: BMC Cardiovasc Disord. 2023 Jan 14;23:19. doi: 10.1186/s12872-022-02904-2 (PMC9840280; doi:10.1186/s12872-022-02904-2)
Supplement: Supplementary file 1 — Additional file 1. Search strategy. [file 12872_2022_2904_MOESM1_ESM.pdf]

Medline

#	Query	Results
1	(MH "Out-of-Hospital Cardiac Arrest") OR TI ("out-of-hospital cardiac arrest" or "out of hospital cardiac arrest") OR AB ("out-of-hospital cardiac arrest" or "out of hospital cardiac arrest")	5,551
2	(MH "Cardiopulmonary Resuscitation") OR TI ("cardiopulmonary resuscitat*" OR "cardio pulmonary resucitat*" or "CPR" or "CPR training") OR AB ("cardiopulmonary resuscitat*" OR "cardio pulmonary resucitat*" or "CPR" or "CPR training")	24,994
3	(MH "Heart Arrest") OR TI ("heart arrest" OR "cardiac arrest") OR AB ("heart arrest" OR "cardiac arrest")	42,883
4	(MH "Heart Massage") OR TI ("heart massage" OR "cardiac massage" OR "chest compression*") OR AB ("heart massage" OR "cardiac massage" OR "chest compression*")	6,263
5	S1 OR S2 OR S3 OR S4	58,002
6	TI (bystander* or layperson* or lay person* or layman or laymen or lay people or laypeople or citizen* or volunteer* or public or "member* of the public" or "general public" or "lay rescuer*" or "layrescuer" or "lay responder*" or "responder*" or "student*" or "pupil*" or "family member*") OR AB (bystander* or layperson* or lay person* or layman or laymen or lay people or laypeople or citizen* or volunteer* or public or "member* of the public" or "general public" or "lay rescuer*" or "layrescuer" or "lay responder*" or "responder*" or "student*" or "pupil*" or "family member*")	931,403
7	S5 AND S6	5,036
8	limit 7 to humans	**4,395**

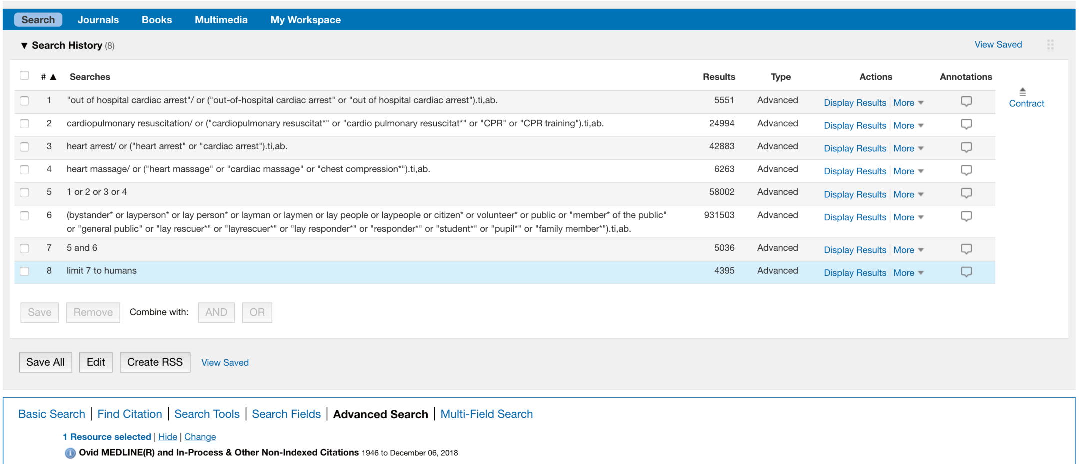

CINAHL

#	Query	Results
1	TI ("out-of-hospital cardiac arrest" or "out of hospital cardiac arrest") OR AB ("out-of-hospital cardiac arrest" or "out of hospital cardiac arrest")	2,818
2	((MH "Resuscitation, Cardiopulmonary") or TI("cardiopulmonary resuscitat*" or "cardio pulmonary resucitat*" or "CPR" or "CPR training") or AB ("cardiopulmonary resuscitat*" or "cardio pulmonary resucitat*" or "CPR" or "CPR training"))	14,572
3	((MH "Heart Arrest") or (TI ("heart arrest" or "cardiac arrest") or AB ("heart arrest" or "cardiac arrest"))	17,552
4	((MH "Heart Massage") or (TI ("heart massage" or "cardiac massage" or "chest compression*") or AB ("heart massage" or "cardiac massage" or "chest compression*"))	2,008
5	S1 OR S2 OR S3 OR S4	25,622
6	(MH "Bystander CPR")	653
7	TI (bystander* or layperson* or lay person* or layman or laymen or lay people or laypeople or citizen* or volunteer* or public or "member* of the public" or "general public" or "lay rescuer*" or "layrescuer" or "lay responder*" or "responder*" or "student*" or "pupil*" or "family member*") OR AB (bystander* or layperson* or lay person* or layman or laymen or lay people or laypeople or citizen* or volunteer* or public or "member* of the public" or "general public" or "lay rescuer*" or "layrescuer" or "lay responder*" or "responder*" or "student*" or "pupil*" or "family member*")	339,087
8	S6 OR S7	339,475
9	S5 AND S8	3,009
10	Limit 9 to Human	**1,469**

CINAHL Screenshot

Select / deselect all | Search with AND | Search with OR | Delete Searches | Refresh Search Results

Search ID#	Search Terms	Search Options	Actions
S10	S5 AND S8	Limiters - Human<br>Search modes - Find all my search terms	View Results (1,469) View Details Edit
S9	S5 AND S8	Search modes - Find all my search terms	View Results (3,009) View Details Edit
S8	S6 OR S7	Search modes - Find all my search terms	View Results (339,475) View Details Edit
S7	TI ((bystander* or layperson* or lay person* or layman or laymen or lay people or laypeople or citizen* or volunteer* or public or "member* of the public" or "general public" or "lay rescuer*" or "layrescuer" or "lay responder*" or "responder*" or "student*" or "pupil*" or "family member*")) OR AB ((bystander* or layperson* or lay person* or layman or laymen or lay people or laypeople or citizen* or volunteer* or public or "member* of the public" or "general public" or "lay rescuer*" or "la ...	Search modes - Find all my search terms	View Results (339,087) View Details Edit
S6	(MH "Bystander CPR")	Search modes - Find all my search terms	View Results (653) View Details Edit
S5	S1 OR S2 OR S3 OR S4	Search modes - Find all my search terms	View Results (25,622) View Details Edit
S4	(MH "Heart Massage") OR TI (("heart massage" or "cardiac massage" or "chest compression*")) OR AB (("heart massage" or "cardiac massage" or "chest compression*"))	Search modes - Find all my search terms	View Results (2,008) View Details Edit
S3	(MH "Heart Arrest") OR TI (("heart arrest" or "cardiac arrest")) OR AB (("heart arrest" or "cardiac arrest"))	Search modes - Find all my search terms	View Results (17,552) View Details Edit
S2	(MH "Resuscitation, Cardiopulmonary") OR TI (("cardiopulmonary resuscitat*" or "cardio pulmonary resucitat*" or "CPR" or "CPR training")) OR AB (("cardiopulmonary resuscitat*" or "cardio pulmonary resucitat*" or "CPR" or "CPR training"))	Search modes - Find all my search terms	View Results (14,572) View Details Edit
S1	TI (("out-of-hospital cardiac arrest" or "out of hospital cardiac arrest")) OR AB (("out-of-hospital cardiac arrest" or "out of hospital cardiac arrest"))	Search modes - Find all my search terms	View Results (2,818) View Details Edit

PsycInfo

#	Query	Results
1	TI ("out-of-hospital cardiac arrest" or "out of hospital cardiac arrest") OR AB ("out-of-hospital cardiac arrest" or "out of hospital cardiac arrest")	52
2	DE („CPR") or TI („cardiopulmonary resuscitat*" OR "cardio pulmonary resucitat*" or "CPR" or "CPR training") or AB ("cardiopulmonary resuscitat*" OR "cardio pulmonary resucitat*" or "CPR" or "CPR training")	900
3	TI ("heart arrest" or "cardiac arrest") or AB ("heart arrest" or "cardiac arrest")	780
4	TI ("heart massage" or "cardiac massage" or "chest compression*") OR AB ("heart massage" or "cardiac massage" or "chest compression*")	51
5	S1 OR S2 OR S3 OR S4	1,590
6	TI (bystander* or layperson* or lay person* or layman or laymen or lay people or laypeople or citizen* or volunteer* or public or "member* of the public" or "general public" or "lay rescuer*" or "layrescuer" or "lay responder*" or "responder*" or "student*" or "pupil*" or "family member*") OR AB (bystander* or layperson* or lay person* or layman or laymen or lay people or laypeople or citizen* or volunteer* or public or "member* of the public" or "general public" or "lay rescuer*" or "layrescuer" or "lay responder*" or "responder*" or "student*" or "pupil*" or "family member*")	734,157
7	S5 AND S6	277
8	limit 7 to human	**271**

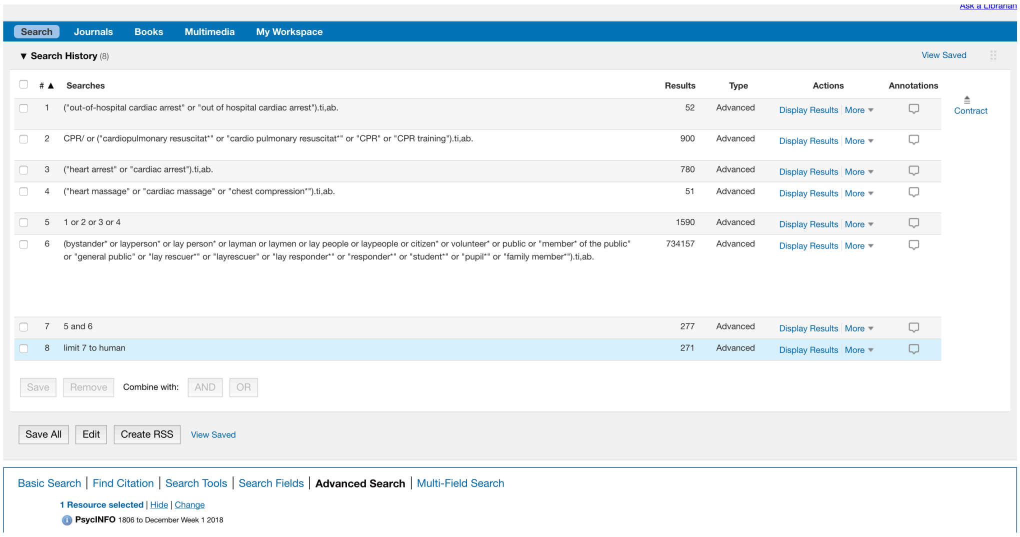

Embase

#	Query	Results
1	"out of hospital cardiac arrest"/ or ("out-of-hospital cardiac arrest" or "out of hospital cardiac arrest").ti,ab.	9,971
2	resuscitation/ or ("cardiopulmonary resuscitat*" or "cardio pulmonary resuscitat*" or "CPR" or "CPR training").ti,ab.	115,884
3	heart arrest/ or ("heart arrest" or "cardiac arrest").ti,ab.	79,489
4	heart massage/ or ("heart massage" or "cardiac massage" or "chest compression*").ti,ab.	9,471
5	1 or 2 or 3 or 4	168,568
6	(bystander* or layperson* or lay person* or layman or laymen or lay people or laypeople or citizen* or volunteer* or public or "member* of the public" or "general public" or "lay rescuer*" or "layrescuer" or "lay responder*" or "responder*" or "student*" or "pupil*" or "family member*").ti,ab.	1,267,023
7	5 and 6	11,819
8	limit 7 to human	10,494

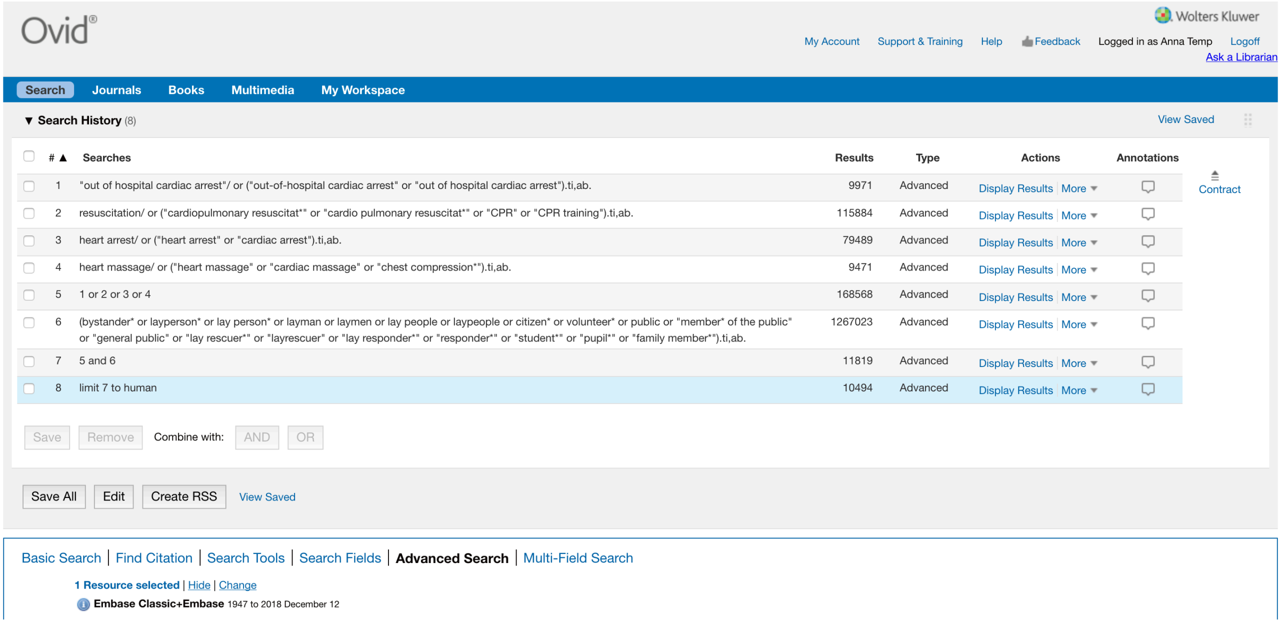

# ▲	Searches	Results	Type	Actions	Annotations
1	"out of hospital cardiac arrest"/ or ("out-of-hospital cardiac arrest" or "out of hospital cardiac arrest").ti,ab.	9971	Advanced	Display Results | More ▾	
2	resuscitation/ or ("cardiopulmonary resuscitat*" or "cardio pulmonary resuscitat*" or "CPR" or "CPR training").ti,ab.	115884	Advanced	Display Results | More ▾	
3	heart arrest/ or ("heart arrest" or "cardiac arrest").ti,ab.	79489	Advanced	Display Results | More ▾	
4	heart massage/ or ("heart massage" or "cardiac massage" or "chest compression*").ti,ab.	9471	Advanced	Display Results | More ▾	
5	1 or 2 or 3 or 4	168568	Advanced	Display Results | More ▾	
6	(bystander* or layperson* or lay person* or layman or laymen or lay people or laypeople or citizen* or volunteer* or public or "member* of the public" or "general public" or "lay rescuer*" or "layrescuer" or "lay responder*" or "responder*" or "student*" or "pupil*" or "family member*").ti,ab.	1267023	Advanced	Display Results | More ▾	
7	5 and 6	11819	Advanced	Display Results | More ▾	
8	limit 7 to human	10494	Advanced	Display Results | More ▾	
